# Metabolic profiling of attached and detached metformin and 2-deoxy-D-glucose treated breast cancer cells reveals adaptive changes in metabolome of detached cells

**DOI:** 10.1038/s41598-021-98642-0

**Published:** 2021-11-01

**Authors:** Jernej Repas, Elmar Zügner, Boris Gole, Maruša Bizjak, Uroš Potočnik, Christoph Magnes, Mojca Pavlin

**Affiliations:** 1 Institute of Biophysics, Faculty of Medicine, University of Ljubljana, Ljubljana, Slovenia; 2 Joanneum Research Health – Institute for Biomedicine and Health Sciences, Graz, Austria; 3 Center for Human Molecular Genetics and Pharmacogenomics, Medical Faculty, University of Maribor, Maribor, Slovenia; 4 Group for Nano- and Biotechnological Applications, Faculty of Electrical Engineering, University of Ljubljana, Ljubljana, Slovenia; 5 Present Address: Pharmacy Institute, Faculty of Pharmacy, University of Ljubljana, Ljubljana, Slovenia; 6 Laboratory for Biochemistry, Molecular biology and Genomics, Faculty for Chemistry and Chemical Engineering, University of Maribor, Maribor, Slovenia

**Keywords:** Biochemistry, Cancer

## Abstract

Anchorage-independent growth of cancer cells in vitro is correlated to metastasis formation in vivo. Metformin use is associated with decreased breast cancer incidence and currently evaluated in cancer clinical trials. The combined treatment with metformin and 2-deoxy-D-glucose (2DG) in vitro induces detachment of viable MDA-MB-231 breast cancer cells that retain their proliferation capacity. This might be important for cell detachment from primary tumors, but the metabolic changes involved are unknown. We performed LC/MS metabolic profiling on separated attached and detached MDA-MB-231 cells treated with metformin and/or 2DG. High 2DG and metformin plus 2DG altered the metabolic profile similarly to metformin, inferring that metabolic changes are necessary but not sufficient while the specific effects of 2DG are crucial for detachment. Detached cells had higher NADPH levels and lower fatty acids and glutamine levels compared to attached cells, supporting the role of AMPK activation and reductive carboxylation in supporting anchorage-independent survival. Surprisingly, the metabolic profile of detached cells was closer to untreated control cells than attached treated cells, suggesting detachment might help cells adapt to energy stress. Metformin treated cells had higher fatty and amino acid levels with lower purine nucleotide levels, which is relevant for understanding the anticancer mechanisms of metformin.

## Introduction

In the last two decades, metabolic alterations have been recognized as an emerging hallmark of cancer cells, providing the potential for use of metabolic drugs in cancer^1–3^. The antidiabetic drug metformin has been associated with decreased incidence of several cancer types including breast cancer^4^. The use of metformin in cancer is being evaluated in several clinical trials (reviewed in^5^). NADH oxidase is considered the main target of metformin in cancer cells^6^, but the precise mechanisms of action are still debated^7,8^ (reviewed in^9^). A combination of metformin and inhibitors of glycolysis was suggested as a possible therapeutic approach^10^ and a synergistic effect with 2-deoxy-D-glucose (2DG) on cancer cell proliferation was demonstrated in vitro^11–13^. However, we observed that treating breast cancer cells with metformin and physiologically relevant concentration of 2DG induced detachment of viable cells that retained their proliferation capacity following reseeding^14^. This effect might be relevant for metastasis formation, where cell detachment from the invasion front is the crucial step in the early stages^15,16^. Following detachment, cancer cells must avoid anoikis—programed cell death in the detached state. Survival in anchorage-independent conditions in vitro is therefore closely correlated to metastasis formation in vivo^17,18^. The anchorage-independent mRNA expression signature also correlates with the aggressiveness of cancer lines in vivo^17^. The MDA-MB-231 triple negative breast cancer cell line is capable of growth in anchorage-independent conditions and has the ability to form metastases in animal models^17,19,20^, making it an ideal model to understand the mechanisms behind the cell detachment.

In recent years, metabolomics has become a potent tool for mechanistic studies of cancer metabolism^21^. Several metabolomics studies have identified the metabolic fingerprint of metformin treated cancer cells in vivo and in vitro^22–32^. However, only a few studies investigated the effect of metformin on the breast cancer cell metabolome^22,23,32^. Previous metabolomics studies of 2DG on cancer cells noted a decrease in glycolytic intermediates downstream of phosphoglucose isomerase, accompanied by a compensatory increase in pentose phosphate pathway (PPP) metabolites^33–35^. Additionally, 2DG inhibits protein mannosylation, confirmed by the competitive levels of UDP-2DG in the metabolome^33,36^. Other studies also suggest that 2DG might affect the urea cycle, one-carbon and purine metabolism, amino acids, biogenic amines and lipids^33,35,37,38^. However, up to date, the combined effects of metformin and 2DG on the cell metabolome have not been elucidated.

It is also still unknown how the population of cells that detached after metformin and 2DG treatment differ metabolically from the cells that remain attached. Anchorage-independent growth induces several cell-type and condition specific metabolic changes, including AMPK activation, altered glucose uptake, increased flux through the PPP and alterations in tricarboxylic acid (TCA) cycle towards reductive carboxylation^17,18,39–41^. However, few studies have focused specifically on the metabolome of the cells in the anchorage-independent conditions^42,43^. Understanding the metabolic changes responsible for detachment of viable cancer cells might help discern the role of metabolism in driving early stages of metastasis formation like tumor budding. Conversely, identifying the metabolic differences of attached and detached populations could help us better understand the metabolic changes involved in avoiding anoikis and surviving in anchorage-independent state.

In the present study, we performed a metabolome analysis in MDA-MB-231 triple negative breast cancer cells treated with metformin, 2DG and their combination. We separately analyzed attached and detached cell populations for different treatments to identify alterations that support detachment and enable anchorage-independent growth of breast cancer cells. Detachment inducing conditions caused a change in the metabolic profile similar to metformin alone. This indicates that metabolic changes are necessary but not sufficient for viable cell detachment, which requires the specific effect of 2DG. Surprisingly, the metabolic profile of detached cells was closer to control cells than attached treated cells, suggesting cell detachment might help cells adapt to energy stress. Finally, we observed marked changes in the metabolic profile of metformin treated MDA-MB-231 cells including changes in fatty and amino acid levels.

## Results

### Metabolic profiling of the attached and detached cell populations

**Figure 1:**
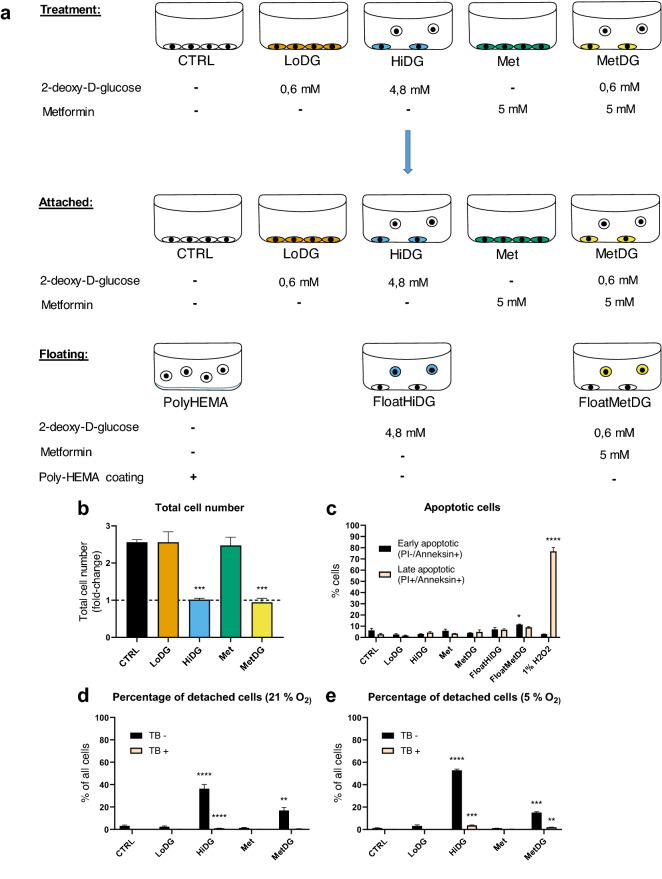
Schematic representation of the treatments (**a**), proliferation (**b**) and viability (**c**) of cell for metabolic profiling. MDA-MB-231 cells were treated for 48 h with metformin and/or 2-deoxy-D-glucose (2DG) with daily medium change. For treatments that induce cell detachment, attached and detached populations were harvested and analyzed separately. Untreated cells grown on poly-HEMA were used as an additional control for floating cells (**a**). Floating and attached cells were counted separately with Countess cell counter and the total cell number (attached + floating) per treatment normalized to the number of seeded cells is presented. The mean ± SEM is shown for three independent experiments (**b**). Cell viability was determined with annexin/PI staining using flow cytometry. The percentage of early apoptotic (annexin+/PI−) and late apoptotic (annexin+/PI+) cells is shown as mean ± SEM for two independent experiments. 1% H2O2 treatment for 1 h was used as positive control (**c**). Cell detachment was separately characterized at atmospheric (21%) and physiological (5%) O2 levels. Cells were treated for 48 h either at 21% O_2_ with daily medium change (**d**) or in a surplus of medium at 5% O_2_ (**e**) and the number of attached and detached cells counted with Trypan blue staining. The mean percentage of detached cells ± SEM is shown for three to six (**d**) or two (**e**) independent experiments. CTRL—attached untreated control cells, LoDG—0.6 mM 2DG treated attached cells, HiDG—attached 4.8 mM 2DG treated cells, Met—attached 5 mM metformin treated cells, MetDG—attached 5 mM metformin + 0.6 mM 2DG treated cells, FloatHiDG—floating detached 4.8 mM 2DG treated cells, FloatMetDG—floating detached 5 mM metformin + 0.6 mM 2DG treated cells, poly-HEMA—floating untreated cells grown on poly-HEMA. *p < 0.05, **p < 0.01, ***p < 0.001.

For the attached population, MDA-MB-231 cells were cultivated on Lumox membranes and treated for 48 h with either drug-free medium (CTRL), 0.6 mM 2DG (LowDG), 4.8 mM 2DG (HiDG), 5 mM metformin (Met) or 5 mM metformin and 0.6 mM 2DG (MetDG) (Fig. 1). For the detached population, MDA-MB-231 cells were cultivated and treated with metformin + 2DG (FloatMetDG) and 4.8 mM 2DG (FloatHiDG). MDA-MB-231 cells grown on poly-hydroxyethylmethacrylate (polyHEMA) to prevent cell attachment were used as a control for the detached population. Metabolic profiling was performed on the eight sample groups (attached population: CTRL, LowDG, HiDG, Met, MetDG; detached population: FloatMetDG, FloatHiDG, polyHEMA) with a total of 99 metabolites (53 MVA_UVA, 46 UVA; Supplementary Table S1) usable for statistical analysis.

The effects of metformin and 2DG treatment on cell proliferation and viability were determined in a separate experiment using direct cell counting for total cell number (Fig. 1b) and annexin/PI staining for the percentage of dead and apoptotic cells in the samples (Fig. 1c). The total cell number was unchanged compared to CTRL in both LoDG and Met, while the total cell number (floating plus attached cells) was significantly lower in both HiDG and MetDG, remaining essentially unchanged from the start of the treatment (Fig. 1b). About 10% of cells stained positive for either annexin and/or PI in CTRL, and this percentage was essentially unchanged in any of the samples with attached cells. There was a weak trend towards higher percentage of dead cells in FloatHiDG and FloatMetDG (*p* > 0.05) and the percentage of apoptotic cells was significantly higher in FloatMetDG compared to CTRL (*p* < 0.05), but in all cases, the percentage of dead cells was under 10%.

To verify whether the phenomenon of metformin and 2DG induced cell detachment is also present at physiological oxygen levels, we characterized cell detachment at atmospheric (21%) and in vivo physiological (5%) oxygen concentration. We found that the percentage of live detached cells was significantly increased compared to CTRL in HiDG and MetDG regardless of oxygen concentration (Fig. 1d,e), while other treatments did not induce significant detachment. The percentage of detached cells was in the range of 15 to 20% for MetDG at both oxygen concentrations. For HiDG, the percentage of live detached cells was somewhat higher at 5% oxygen compared to 21% (53% versus 36%).

### The effect of metformin and 2DG treatment on the metabolic profile of MDA-MB-231 cells

**Figure 2:**
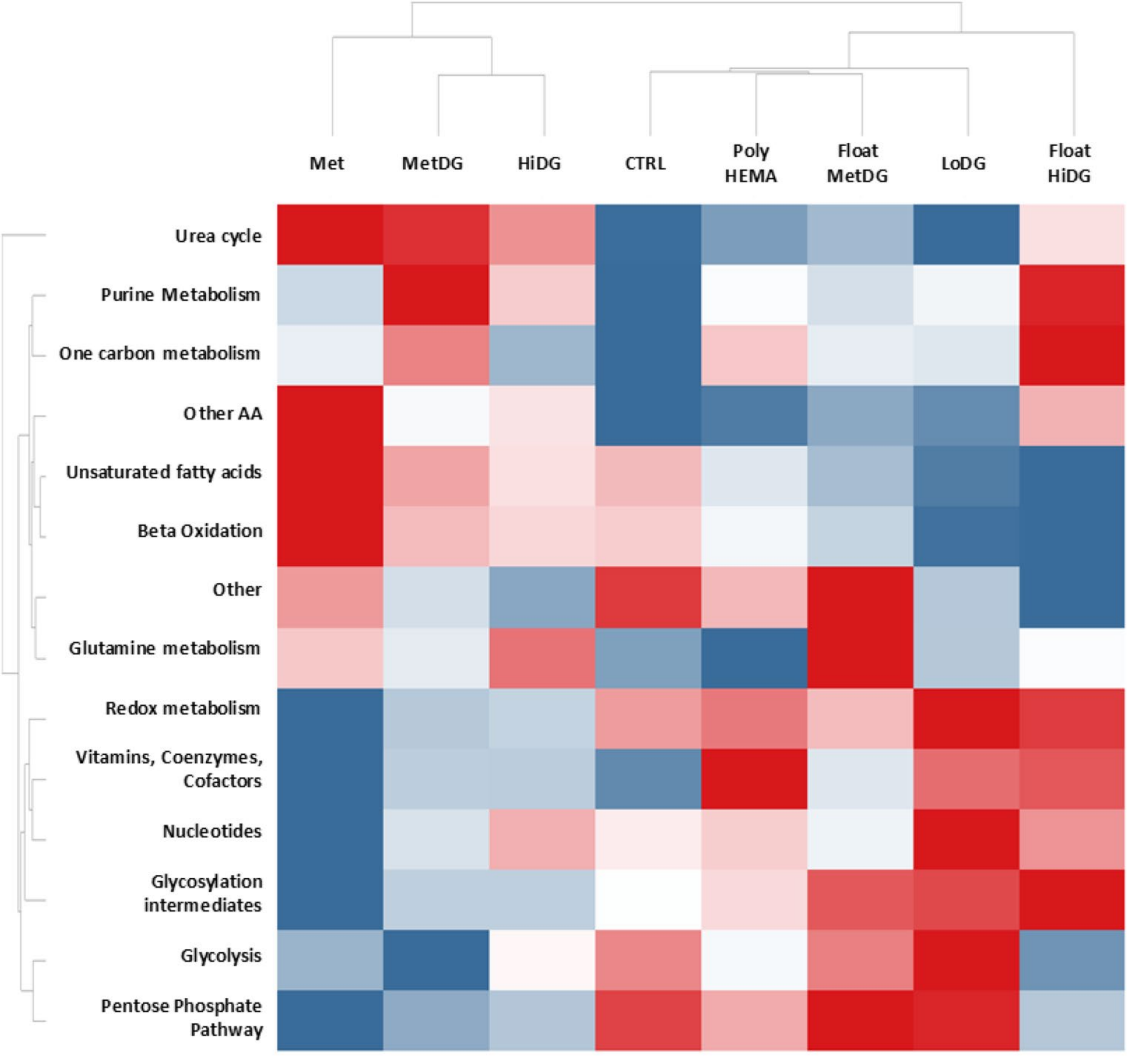
Heat map of selected metabolite levels according to major metabolic pathways. Measured metabolites were classified according to their primary metabolic pathways (refer to Supplementary Table 2). For each pathway and treatment group, the log-median-QC normalized value was used. Red—maximum levels, blue—minimum levels. Met—attached 5 mM metformin treated cells, MetDG—attached 5 mM metformin + 0.6 mM 2DG treated cells, HiDG—attached 4.8 mM 2DG treated cells , CTRL—attached untreated control cells, poly-HEMA—floating untreated cells grown on poly-HEMA, FloatMetDG—floating detached 5 mM metformin + 0.6 mM 2DG treated cells, LoDG—0.6 mM 2DG treated attached cells, FloatHiDG—floating detached 4.8 mM 2DG treated cells.

To characterize the effects of metformin and 2DG on the overall metabolic profile of attached and floating cells, we first performed a heat map analysis using median metabolite levels per treatment group and metabolic pathway (Fig. 2; for metabolite annotation refer to Supplemental Table S2). For attached cells, the automated clustering detected the highest dissimilarities between Met and CTRL. Urea cycle, purine and one-carbon metabolism, saturated and unsaturated FA, as well as glutamine and other AA were increased in Met compared to CTRL. Glycolysis, PPP, redox metabolites, vitamins, coenzymes and cofactors, nucleotides and glycosylation intermediates were decreased. The MetDG showed a very similar pattern to Met but with less pronounced changes compared to the CTRL. The exceptions were greater increase in purine and one-carbon metabolism and a more pronounced decrease in glycolysis metabolites. HiDG was similar to MetDG except for higher levels of nucleotides and slightly lower levels of FA. LoDG was rather distinct from both HiDG and CTRL (except for urea cycle, purine metabolism, one-carbon metabolism and amino acids). FA levels were lower compared to CTRL, while redox metabolites, nucleotides, glycolysis, PPP metabolites and glycosylation intermediates were increased. Heat map clustering using individual metabolites (Supplementary Fig. S1) and principal component analysis (Supplementary Fig. S2) gave very similar results for attached cells, with the exception of LoDG clustering closer to CTRL.

To determine the metabolic alterations specific to floating cells, we then compared the metabolic profiles of floating and attached cells (Fig. 2). FloatMetDG was separated from MetDG and showed closer relations to CTRL. Levels of some metabolites in FloatMetDG sample were between CTRL and MetDG (urea cycle, purine, one-carbon and redox metabolism, some amino acids) while the others were mostly similar to CTRL (glycolysis and PPP metabolites). Unique features of FloatMetDG compared to MetDG were high levels of glutamine related metabolites and glycosylation intermediates, and lower FA levels. PolyHEMA clustered close to CTRL but exhibited elevated levels in vitamins, coenzymes and cofactors and more moderate glycolysis and PPP metabolite levels. The profile of FloatHiDG had some similarities to HiDG, namely in urea cycle, PPP and glycolysis metabolite and nucleotide levels (Fig. 2). On the other hand, purine and one-carbon metabolites were elevated in FloatHiDG compared to HiDG. Saturated and unsaturated FA levels in FloatHiDG were lowered compared to CTRL, while redox metabolites, vitamins, coenzymes and cofactors, and glycosylation intermediates were elevated. Heatmap clustering using individual metabolites instead of classes (Supplementary Fig. S1) gave similar results for FloatMetDG and polyHEMA, while FloatHiDG presented a separate category. The same pattern was observed in PCA (Supplementary Fig. S4).

### The effect of metformin and 2DG treatment on individual metabolites in the attached MDA-MB-231 cells

**Figure 3:**
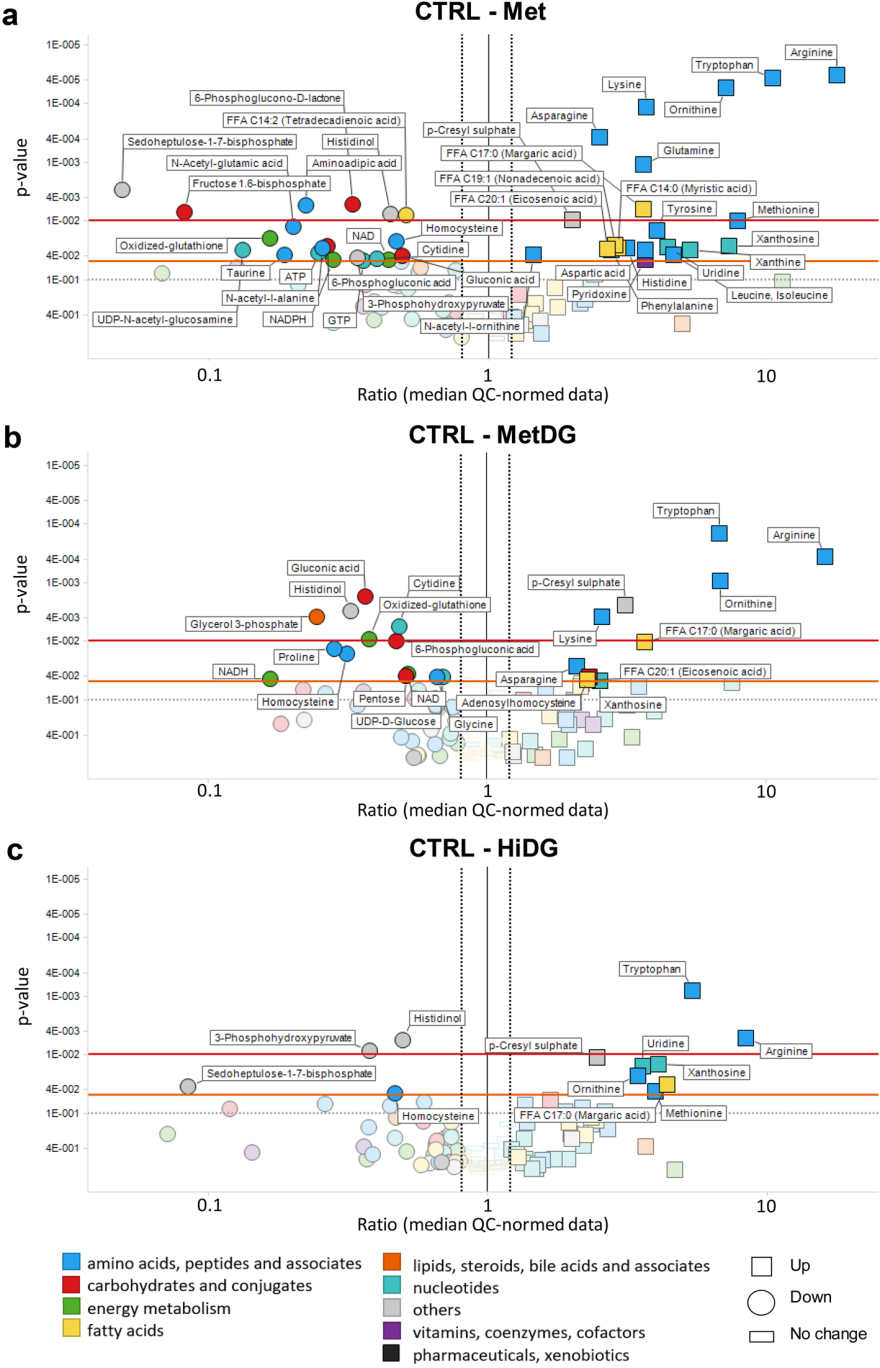
Volcano plots of metabolite levels in the attached cells for Met (**a**), MetDG (**b**) and HiDG (**c**) compared to control. Metabolites on the left side of the black line were decrease while those on the right side of the black line were increased in Met (**a**), MetDG (**b**) or HiDG (**c**) compared to CTRL. Y-axis: *p* values (inverse log scale); x-axis: metabolite ratios (median QC normalized data, log-scaled). Left black dotted line (0.8)—ratio of 0.8; right black dotted line (1.2)—ratio of 1.2. The red line denotes a *p* value of 0.01, the orange line denotes a *p* value of 0.05 and the grey line denotes a *p* value of 0.1. Metabolites are colored according to the HMDB classes. Metabolites with *p* value bellow 0.05 and metabolite ratio > 1 or < 0.8 are named. Metabolites are marked with circle for decrease and square for increase in Met (**a**), MetDG (**b**) or HiDG (**c**) compared to CTRL.

To identify which individual metabolites were most significantly affected by metformin and/or 2DG treatment, we compared individual metabolite levels between treatment groups with analysis of variance (ANOVA). Met showed significant differences in metabolites compared to CTRL (Fig. 3a). The amino acids arginine, ornithine, tryptophan, lysine, glutamine and asparagine were higher in Met compared to CTRL (*p* < 0.01). Methionine, tyrosine, histidine, aspartic acid, phenylalanine, and combined pool of leucine and isoleucine were also higher in Met (*p* < 0.05). Uridine, xanthosine and xanthine were higher in Met, whereas cytidine was lowered compared to CTRL (*p* < 0.05). Redox cofactors NAD and NADPH, oxidized glutathione as well as homocysteine, ATP and GTP were significantly reduced in Met (*p* < 0.05). Fructose-1,6-bisphosphate was decreased (*p* < 0.01) while other glycolysis intermediates showed no significant changes. A significant decrease in all measured PPP metabolites as well as a glycosylation intermediate (UDP-N-acetyl-glucosamine) was observed. Significant increases compared to CTRL were also detected for some FA.

Metabolic changes in MetDG compared to CTRL (Fig. 3b) were similar to Met for the amino acids and energy cofactors. Among PPP metabolites, only 6-phosphogluconic acid was lowered (*p* < 0.01) compared to CTRL. Adenosylhomocysteine levels were higher (*p* < 0.05) while homocysteine, proline and glycine levels were lower (*p* < 0.05) compared to CTRL. There were only two significantly increased FA compared to CTRL. Fewer metabolites were significantly changed compared to CTRL in MetDG than in Met. Comparing MetDG to Met only showed few significant changes (Supplementary Fig S3). Levels of inosine, guanosine, and aminoadipic acid were increased (*p* < 0.05), while proline, valine, hexose (*p* < 0.05) and pentose (*p* < 0.01) were decreased.

HiDG showed similar increases to MetDG for the AA tryptophan and arginine (*p* < 0.01), as well as methionine and ornithine (*p* < 0.05) compared to CTRL (Fig. 3c). Homocysteine and sedoheptulose-1,7-bisphosphate levels were decreased (*p* < 0.05), whereas uridine and xanthosine levels were increased (*p* < 0.05) compared to CTRL. No significant changes for HiDG in FA (with the exception of margaric acid), ATP, NADPH, NADH or glycosylation intermediates were detected compared to CTRL. In LoDG, only adenosylhomocysteine and tryptophan levels were increased (*p* < 0.05) compared to CTRL (Supplementary Fig. S3).

### Metabolite levels in FloatMetDG and FloatHiDG

**Figure 4:**
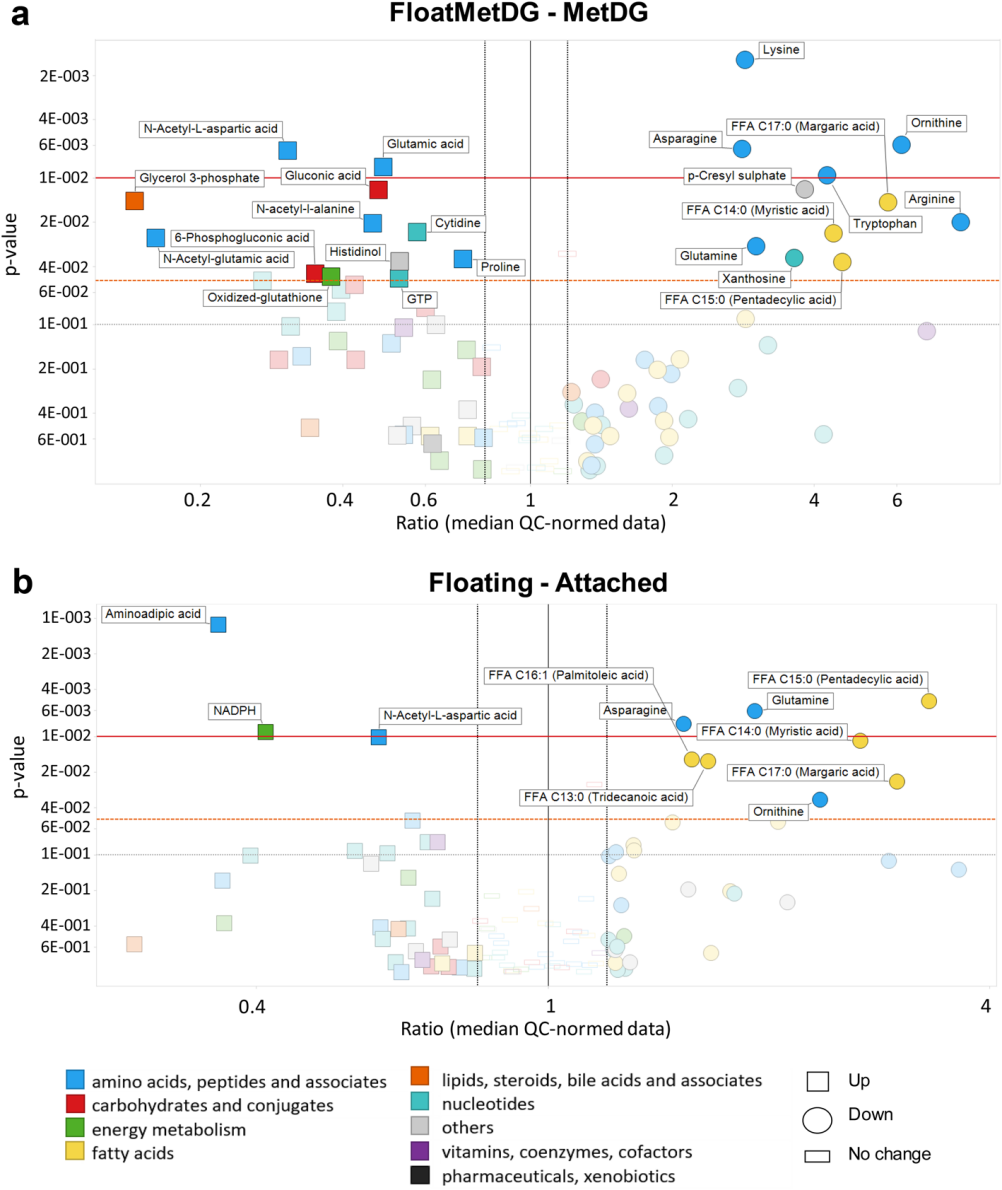
Metabolic differences between floating and attached cell populations for FloatMetDG versus MetDG (**a**) or overall floating versus attached cells (**b**). Metabolites on the left side of the black line were increased while those on the right side of the black line were decreased in FloatMetDG compared to MetDG (**a**) or overall in all floating compared to all attached cells (**b**). Y-axis: *p* values (inverse log-scale); x-axis: metabolite ratios (median QC normalized data, log-scaled). Axis scale is different for (**a**) and (**b**) Left black dotted line (0.8)—ratio of 0.8; right black dotted line (1.2)—ratio of 1.2. The red horizontal line denotes a *p* value of 0.01, the orange line denotes a *p* value of 0.05 and the grey line denotes a *p* value of 0.1. Metabolites are colored according to the HMDB classes. Metabolites with *p* value bellow 0.05 and metabolite ratio > 1.2 or < 0.8 are named. Metabolites are marked with a square for increase and a circle for decrease in floating compared to attached cells.

To identify individual metabolites responsible for the altered metabolic profile of floating cell populations, we performed ANOVA analysis for metabolite levels. Compared to MetDG, FloatMetDG (Fig. 4a) had lower levels of several AA including glutamine (*p* < 0.05), lysine and ornithine (*p* < 0.01). Levels of glutamic acid (*p* < 0.01) and proline (*p* < 0.05), 6-phosphogluconic acid, oxidized glutathione, and glycerol-3-phosphate levels were increased (*p* < 0.05). FloatMetDG showed significant increases in acetylated AA N-acetyl-aspartic acid (*p* < 0.05), N-acetyl-alanine and N-acetyl-glutamic acid (*p* < 0.05) levels, while some FA were significantly decreased. FloatHiDG (Supplementary Fig. S5) had lower levels of hexose (*p* < 0.05) as well as glutamine and tridecanoic acid (*p* < 0.05) compared to HiDG. Levels of NADPH and aminoadipic acid (*p* < 0.01) as well as ATP and UDP-N-acetyl-glucosamine (*p* < 0.05) were increased in FloatHiDG compared to HiDG. Compared to CTRL, only six metabolites were significantly changed in polyHEMA. Levels of tryptophan (*p* < 0.01) and xanthosine (*p* < 0.05) were increased, while proline (*p* < 0.01), cytidine, taurine, and fructose-1,6-bisphosphate (*p* < 0.05) were decreased.

Comparing the floating population group (FloatMetDG, FloatHiDG and poly-HEMA) to the attached population group (CTRL, LoDG, HiDG, Met and MetDG) in the ANOVA (Fig. 4b) showed significantly lower levels of AAs asparagine and glutamine (*p* < 0.01), ornithine, histidine and lysine (*p* < 0.05), as well as several FA, in the floating cells. Levels of aminoadipic acid and NADPH (*p* < 0.01) were higher in floating cells, as was N-acetyl-aspartic acid (*p* < 0.05).

### Metabolic pathway analysis of energy and redox metabolism

**Figure 5:**
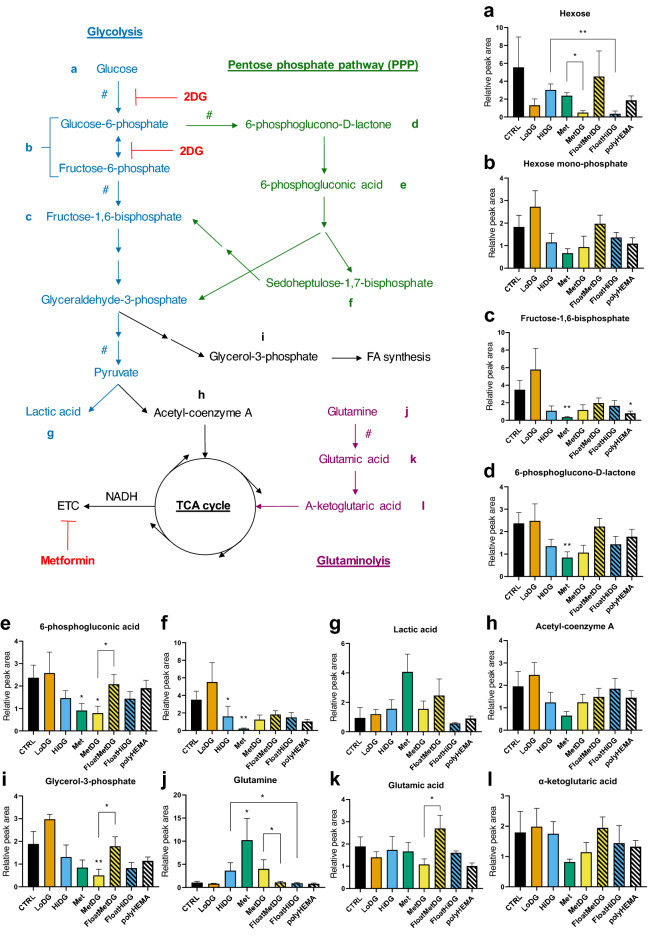
Glycolysis and PPP metabolite levels per treatment groups and their floating populations. Metabolite levels were QC-normalized and mapped to glycolysis and pentose phosphate pathway to show the metabolic reactions in the cell. A single arrow denotes direct conversion in a single reaction and a segmented arrow denotes conversion with multiple step reactions. Rate limiting reactions are marked with #. Samples are color-coded and samples with floating cells are hatched. All graphs show mean relative normalized metabolite peak areas ± standard error (SEM). Please note y-axis scales are different between the plots. *p < 0.05, **p < 0.01 versus CTRL unless otherwise indicated.

To better understand the metabolic effects of metformin and 2DG as well as cell detachment in MDA-MB-231 cells, multivariate analysis of variance (MANOVA) was performed on the metabolites from the most important metabolic pathways (for metabolite classification see Supplementary Table 3), focusing on energy and redox metabolism. HiDG showed significantly changed glycolysis metabolites compared to CTRL (*p* < 0.05). Met showed a similar trend (*p* > 0.05). There were no significant differences between MetDG and either CTRL or Met. In Met, LoDG and MetDG compared to CTRL, hexose showed a trend towards lower levels (*p* > 0.05, Fig. 5). Fructose-1,6-bisphosphate levels were significantly lowered in Met (*p* < 0.01), while HiDG and MetDG showed a similar trend (*p* > 0.05). Mean lactate levels were four times higher in Met compared to CTRL but did not reach significant *p* value. Hexose was suppressed in MetDG compared to Met (*p* < 0.05). MANOVA showed no significant differences between floating and attached populations in glycolysis metabolites. FloatHiDG had lower hexose levels than HiDG (*p* < 0.01) and fructose-1,6-bisphosphate was lowered (*p* < 0.05) in polyHEMA compared to CTRL.

For PPP metabolites, MANOVA showed significant differences in Met compared to CTRL (*p* < 0.05). Compared to CTRL (Fig. 5), 6-phosphogluconic acid was lower in Met and MetDG (*p* < 0.05), while 6-phosphogluconolactone levels were only significantly lowered by Met (*p* < 0.01). Sedoheptulose-1,7-bisphosphate levels were lower in Met (*p* < 0.01) and HiDG (*p* < 0.05) compared to CTRL. Comparing floating and attached cells, MANOVA showed PPP metabolites also differed significantly in polyHEMA compared to CTRL (*p* < 0.05). For individual metabolites, only 6-phosphogluconic acid was higher in FloatMetDG versus MetDG (*p* < 0.05).

MANOVA revealed that FA metabolism was impacted by Met compared to CTRL. Levels of most FA showing a significant difference were higher in Met, MetDG or HiDG compared to CTRL (Fig. 3a). FA metabolism was also significantly changed (*p* < 0.001) between all floating and attached cells, as well as in FloatMetDG versus MetDG (*p* < 0.05) and polyHEMA versus CTRL (*p* < 0.05). Levels of individual FA showing significant differences were lower in floating cells (Fig. 4). Glutamine levels were increased (*p* < 0.01) in Met compared to CTRL (Fig. 5), with MetDG and HiDG showing a similar trend (*p* > 0.05). Both FloatMetDG and FloatHiDG had lower glutamine levels compared to MetDG and HiDG, respectively (*p* < 0.05). Glutamic acid levels were higher in FloatMetDG versus MetDG (*p* < 0.01).

**Figure 6:**
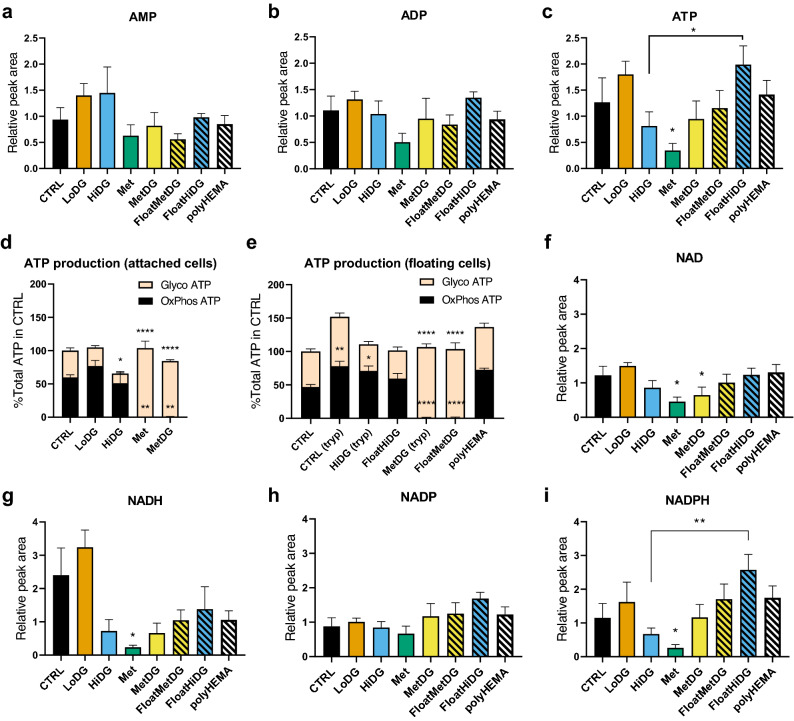
Energy and redox metabolism in MDA-MB-231. Mean relative QC-normalized metabolite peak areas of adenosine phosphates (**a-c**) and redox cofactors (**f–i**) ± SEM are shown from metabolic profiling. (**d, e**) MDA-MB-231 cells were treated for 48 h as indicated. Following treatment, extracellular acidification and oxygen consumption rate were measured and ATP production was obtained according to Seahorse Real-Time ATP Rate Assay protocol. Results are normalized to total ATP production in attached control cells. The mean ± SEM of 3 (**d**) or 4 (2 for poly-HEMA, (**e**)) independent experiments is shown. Please note y-axis scales are different between the plots. *p < 0.05, **p < 0.01. ***p < 0.001, ****p < 0.0001 versus CTRL unless otherwise indicated. In (**d**) and (**e**), significance is marked separately for glycolytic and OxPhos ATP production.

For redox cofactors, Met and MetDG had lower NAD levels compared to CTRL (*p* < 0.05). NADH levels were also lower (*p* < 0.05) in MetDG compared to CTRL (Fig. 6). NADPH levels were lower in Met compared to CTRL (*p* < 0.05). HiDG or LoDG did not significantly change any redox cofactors compared to CTRL. Comparing all floating and attached cells, floating cells had higher levels of NADPH (*p* < 0.01, Fig. 4). NADPH was also elevated in FloatHiDG compared to HiDG (*p* < 0.01).

MANOVA for purine nucleotides (AMP, ADP, ATP, GMP, GDP, GTP) revealed significant changes (*p* < 0.05) in HiDG compared to CTRL. ATP and GTP levels were lower in Met compared to CTRL (*p* < 0.05, Fig. 3 and Fig. 6). MANOVA also showed significant changes in purine for all floating population versus attached cells (*p* < 0.001), specifically in FloatHiDG versus HiDG (*p* < 0.01) and polyHEMA versus CTRL (*p* < 0.05). FloatHiDG had higher ATP levels than HiDG (*p* < 0.05) and FloatMetDG had significantly higher GTP levels compared to MetDG (*p* < 0.05).

### Glycolytic and mitochondrial ATP production

Glycolysis and oxidative phosphorylation (OxPhos) both contributed approximately half of the total ATP production in attached cells (Fig. 6d) as determined by Seahorse Analyser XFe24. LoDG only decreased glycolysis slightly (*p* > 0.05). Glycolytic ATP production was suppressed with HiDG (*p* < 0.05), while the contribution of OxPhos was not changed significantly. The total ATP production was decreased to 65% of CTRL. ATP production from OxPhos was completely suppressed in Met (*p* < 0.0001). The total ATP production was not significantly changed compared to CTRL due to a corresponding increase in glycolytic ATP production. MetDG completely suppressed ATP production from OxPhos (*p* < 0.0001) compared to Met, but also slightly decreased glycolysis (*p* > 0.05). The lack of a more pronounced decrease in OxPhos ATP production with 4.8 mM 2DG treatment can be explained by the metabolic shift to use alternative sources of reducing equivalents (glutamine and fatty acids present in the medium) as well as the incomplete block of glycolysis.

In the floating cells (Fig. 6e), the effects of 2DG and metformin were proportionally similar as in attached cells. Compared to trypsinised control cells, HiDG showed a trend towards lower glycolytic ATP production (*p* > 0.05). MetDG completely suppressed ATP production from OxPhos (*p* < 0.0001), while the total ATP production was about 2/3 of trypsinized control cells. No difference between the FloatHiDG versus trypsinised HiDG or FloatMetDG versus trypsinised MetDG was observable. The oxidative ATP production in trypsinised cells was higher compared to CTRL (*p* < 0.01), and there was a trend towards higher glycolytic and total ATP production (*p* > 0.05). The ATP production rates in polyHEMA were very similar to trypsinized control cells but did not significantly differ from CTRL (*p* > 0.05).

### Transcriptional regulation of energy metabolism and stem-like phenotype of metformin and 2DG treated cells

**Figure 7:**
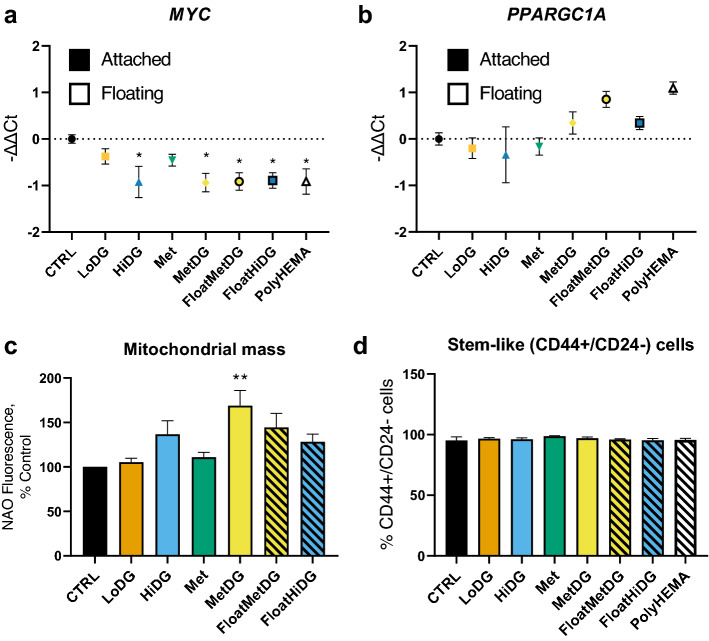
Expression of transcription factors MYC (**a**) and PGC-1α (**b**), mitochondrial mass (**c**) and breast cancer stem cell markers CD44/CD24 (**d**)**.** MDA-MB-231 cells were treated for 48 h (**a**–**c**) or 72 h (**d**), after which cells were collected and RNA was isolated and quantitative real time PCR performed (**a, b**). Mitochondrial mass was determined with flow cytometry using nonyl acridine orange (NAO) staining, (**c**) stem cell markers CD44 and CD24 were measure by flow cytometry (**d**). The mean − ΔΔCt ± SEM is shown for 4 independent experiments (**a, b**) and the mean NAO fluorescence per cell ± SEM (**c**) or percentage of CD44+/CD24− cells ± SEM (**d**) is shown for 3 independent experiments. **p < 0.01 as determined by ANOVA.

To determine effects of treatments and the phenotype of floating cells on the expression of two central transcription factors involved in carcinogenesis, proliferation, stemness (*MYC*) and mitochondrial biogenesis (*PPARGC1A*), quantitative real time PCR was performed (Fig. 7). *MYC* mRNA expression was lower in HiDG, MetDG and all floating cells compared to CTRL (*p* < 0.05) (Fig. 7a). *PPARGC1A* mRNA only increased expression compared to CTRL in FloatMetDG and polyHEMA, but the difference was not significant (*p* > 0.05) (Fig. 7b). The difference was even smaller when comparing floating and attached cells, but mean expression levels were higher in all floating cells (*p* > 0.05). To confirm whether these changes at transcriptional level were also reflected in mitochondrial mass, we also measured the NAO fluorescence using flow cytometry. We found that cells in MetDG had significantly higher mitochondrial mass compared to CTRL (*p* < 0.05) and a similar trend was observed for HiDG (*p* > 0.05), while LoDG and Met had no major effect. Mitochondrial mass was also similar in floating and attached cells (*p* > 0.05). Additionally, we tested whether the detached cells express stemness surface markers for breast cancer stem-like cells (CD44+/CD24−) using flow cytometry. None of the attached or floating cells significantly differed from control in the percentage (> 95%) of stem-like cells (*p* > 0.05) (Fig. 7c).

## Discussion

The first two steps of metastasis formation are the detachment of cells from the primary tumor and the avoidance of anoikis in the anchorage independent conditions^15^. To explore which metabolic changes are potentially responsible for detachment of viable cells, we performed metabolic profiling of MDA-MB-231 breast cancer cells treated with metformin and/or 2DG. We also determined the metabolic differences between the attached cells and detached cells grown in the anchorage independent conditions. Since the observed detachment is reversible and detached floating cells are able to regain normal proliferation rate after reseeding in standard medium^14^, understanding the alterations in metabolic pathways involved can shed light on the mechanisms that induce detachment and enable survival of cells in the detached state. Furthermore, as the mechanisms of anticancer action of metformin are still debated, we also explored the effect of metformin on the metabolic pathways in the MDA-MB-231 cells.

### Metabolic changes induced by metformin and 2DG

Our results indicate that treatments inducing reversible viable cell detachment (metformin + 0.6 mM 2DG (MetDG) and 4.8 mM 2DG alone (HiDG)) cause a notable change in overall metabolic profiles of the attached cells compared to the control cells. The overall metabolic profiles of MetDG and HiDG were very similar despite different mechanisms of action, suggesting the possible existence of specific metabolic alterations conducive to cell detachment. However, the metabolic profiles of MetDG and HiDG were also similar to metformin (Met) treatment, which by itself does not induce cell detachment^14^. Amongst all the samples, the metabolic profile of Met was the most different from CTRL both in terms of median metabolite levels per pathway (Fig. 2) and the number of significantly changed metabolites (Fig. 3). Comparing MetDG to Met, their metabolic profiles were very similar in terms of metabolic pathways (Fig. 2). Both treatments also had a comparable effect on ATP production from OxPhos. LoDG, which also does not induce cell detachment, significantly affected the levels of only two metabolites and had a negligible effect on ATP production, leading to the conclusion that metformin is the primary source of observed metabolic changes in MetDG. Since metformin alone does not induce cell detachment, an altered metabolic profile is not sufficient for this process. Instead, cell detachment seems to be a consequence of specific action of 2DG distinct from glycolysis inhibition, potentiated by metabolic alterations induced by metformin or higher concentrations of 2DG itself.

The very different mechanisms of action of metformin and 2DG, together with the relative similarity of metabolic profiles of MetDG and HiDG raises the question which metabolic features are crucial for the potentiation of the specific effect of 2DG on cell detachment. Both drugs partially inhibit the energy metabolism, making the availability of ATP a potentially crucial parameter. Overall, the total ATP production was unchanged in Met and only moderately decreased in both HiDG and MetDG. Levels of adenine phosphates were similar between these three treatments. This is consistent with previous research where only slight alterations in ATP and AMP levels with 2DG treatment were reported^33^. As previously shown, the AMPK activators alone do not induce cell detachment^14^. Combined with the results shown here, we can conclude that altered cell energy status is one of the most distinguishing features but is not alone sufficient for cell detachment.

Also of note is the effect of detachment inducing treatments on cell proliferation. While proliferation was essentially unchanged in Met or LoDG compared to CTRL, both MetDG and HiDG strongly suppressed proliferation without increasing the fraction of dead cells, consistent with our previous study^14^. While this is in agreement with the proposed “go or grow” paradigm of proliferation and migration (or in this case, cell detachment as the potential first step of the metastatic cascade) being alternative cell fates, this shift is again not entirely obvious from the observed metabolic profiles. The metabolic levels in MetDG and HiDG are, as discussed above, in most cases intermediate between CTRL and Met, which has no major effect on cell proliferation. This means that suppressed proliferation is unlikely to be the main driver for altered metabolite levels compared to CTRL in metformin and 2DG treated cells, but may instead help to partially explain the more modest changes in MetDG and HiDG to those in Met. It is also important to note that proliferation can be suppressed by other treatment combinations (5 mM metformin in medium with 0 g/l glucose), however in this cases not only proliferations is suppressed but large percentage of dead cells was observed^14^. Furthermore, since cell proliferation is a metabolically demanding process, suppressing proliferation would decrease the consumption of nutrients and biosynthetic intermediates, helping to normalize metabolite levels. While this is undoubtedly an important factor, the very different mechanisms of action of metformin and 2DG necessitate a more detailed exploration of their specific effects in inducing the observed metabolic profiles.

As metformin and 2DG inhibit OxPhos and glycolysis, respectively, we expected them to alter metabolite levels and metabolic flux through glycolysis, PPP and TCA cycle. Indeed, the extracellular flux analysis confirmed that metformin and 2DG significantly changed the glycolytic rate, as HiDG significantly lowered glycolysis ATP production, while metformin completely suppressed OxPhos ATP production with a compensatory increase in glycolysis as expected. The effect of LoDG on the glycolytic rate was very modest, both alone or in combination with metformin. However, the glycolysis inhibition by HiDG was not reflected in the levels of glycolytic metabolites, which were similar between Met, MetDG and HiDG. The levels of fructose-1,6-bisphosphate were, for example, only significantly lowered by metformin and showed a similar trend in HiDG and MetDG. MANOVA analysis also confirmed that HiDG, Met and MetDG all affected the metabolites in glycolysis. This is broadly consistent with previous studies and explainable by the role of phosphofructokinase as the rate-limiting step of glycolysis^33^. As 2DG inhibits the two prior reactions catalyzed by hexokinase and phosphoglucose isomerase^44,45^, lower levels of fructose-6-phosphate would lead to even slower production of fructose-1,6-bisphosphate compared to later steps, leading to a depletion of this metabolite^33–35^. The same effect would be achieved by increasing the rate of later reactions more than the activity of the phosphofructokinase or potentially by diverting more pyruvate from the TCA cycle to lactate production, as has been reported for metformin^46^. Since PPP shares its starting substrate with glycolysis and can feed its products back into later stages of glycolysis, changes in the glycolytic rate can also influence the levels of PPP metabolites. Our results confirm this, as HiDG, Met and MetDG all impacted the PPP metabolites with a trend towards lower levels compared to CTRL, which is in agreement with studies on various cancer cell lines^31,33–35^.

As glycolytic and PPP metabolites function as starting substrates for other biosynthetic reactions, their levels directly influence the rate of these reactions. The decreased glycolytic and PPP metabolite levels could therefore impact several metabolic pathways (eg. serine, ribose and nucleotide synthesis, triglyceride and phospholipid synthesis, hexosamine pathway and other glycosylation intermediates) irrespective of actual glycolytic and PPP rates. Significantly lower levels of some metabolites used in these pathways were detected (3-hydroxypyruvate in Met and 4.8 mM 2DG, glycerol-3-phosphate, pentose and UDP-glucose in MetDG, UDP-N-acetylglucosamine in Met), but not all related metabolites were accessible. It is possible that the one of these downstream metabolic changes could be crucial for cell detachment.

Besides glycolysis, a major ATP source is mitochondrial OxPhos, which is linked to the TCA cycle fueled by acetyl-coenzyme A derived from pyruvate or fatty acid oxidation. In basal conditions, MDA-MB-231 cells obtain about half of ATP from OxPhos, which was completely suppressed in Met and MetDG (Fig. 6). We also detected important changes in the glutamine metabolism which can replenish the TCA cycle intermediates. While metformin treatment can lead to reductive carboxylation of glutamine derived α-ketoglutarate^47^, glutamine levels in our study were vastly increased in Met (Fig. 5) suggesting decreased conversion of glutamine to α-ketoglutarate. This is in agreement with reports of metformin inhibiting glutaminase^48^ and decreasing the mitochondrial conversion of glutamine to citrate^8^. We observed a similar trend in MetDG and HiDG. The higher levels of FA in Met, were consistent with the study by Lord et al. demonstrating FA accumulation^32^. This suggests that FA oxidation was suppressed in Met, since the study by Griss et al. demonstrating decreased FA synthesis with metformin treatment in lung cancer cells makes increased FA synthesis an unlikely explanation^8^. The trend for FA was less pronounced in MetDG and especially HiDG, which is in agreement with unchanged OxPhos ATP production in HiDG compared to control. Previous research also supports the lack of TCA perturbation by 2DG when alternative TCA substrates are available^33,34^, although some studies did find an effect of 2DG on lipids in keratinocytes and oral squamous carcinoma^37^. Overall, the vastly different OxPhos in MetDG and HiDG means oxidative metabolism is unlikely to directly drive cell detachment.

We expected the redox balance in Met to be shifted towards reduced cofactors, especially NADH, as observed in several cancer types and cell lines^24–26,31^. Our results show that Met significantly decreased both NAD and NADPH while also showing a trend towards decreased NADH. This discrepancy could be accredited to cancer type or cell line characteristics, as the studies mentioned did not use breast cancer cells. MetDG had a similar effect on NAD and NADH, but not NADPH levels. HiDG showed a trend towards lower NADH levels but did not significantly change NAD, NADPH or NADP, which is in agreement with the study by Urakami et al. using endometrial cancer cells^33^. Despite altered PPP metabolite levels, HiDG and MetDG cells were therefore able to maintain a similar ROS balance. As NADPH levels were very low in Met where cell detachment is not observed, the importance of the redox balance for cell detachment seems plausible, in accordance with previous work outlining its importance in anchorage-independent growth^39,49^.

Additionally, the redox balance also affects the synthesis of some metabolites, like aspartate^50^ and glycerol-3-phosphate. We found significantly increased aspartate and decreased glycerol-3-phosphate levels in Met, consistent with NAD and NADH levels. This differs from some of the previous studies^24,26^, even though one did report increased aspartate levels in a high glucose medium^25^. Several nucleotide and nucleoside levels were also significantly changed in Met, MetDG and HiDG. Besides precursors from PPP and redox balance, nucleotide synthesis is also dependent on one carbon units and amino acids (AA). We observed changes in one carbon metabolism in MetDG and LoDG, although only HiDG significantly affected the one carbon metabolism as a whole (Supplementary Fig. S6). Proline and glycine levels were significantly decreased in MetDG, while most other amino acid levels showed a trend towards elevated levels in Met, MetDG and HiDG (Fig. 3, Supplementary Fig S7) possibly reflecting the decreased consumption due to suppressed proliferation. However, we have previously found metformin did not affect the proliferation of MDA-MB-231 cells when there is sufficient glucose^51^, despite impacting AA levels the most. The increased AA levels are therefore probably the result of either increased uptake or decreased use in other reactions.

Overall, the similar changes in metabolite levels seen in Met, MetDG and HiDG do not support the hypothesis of a specific metabolomics phenotype that induces cell detachment. The similar metabolic profiles of these treatments likely stems from their similar effect on decrease in total ATP production, glycolytic and PPP metabolite levels and to some extent redox cofactors. On the other hand, cell detachment occurs due to some specific effects of 2-DG, downstream of major metabolic alterations, as evidenced by the potentiating effect of metformin. In addition to inhibiting glycolysis, 2DG has been shown to inhibit protein mannosylation^33,36^, induce ER stress^52,53^ and the unfolded protein response^54^ , and to activate AMPK independent of AMP levels, which could affect cell detachment either by directly modifying the characteristics of surface proteins or by altering cell signaling pathways involved in cell attachment. In the present study, we could not find conclusive evidence in the metabolic profile for either of these possibilities, so further studies will be needed to investigate the precise mechanism of 2DG induced cell detachment.

### Metabolic changes in floating cells

Survival of cancer cells in anchorage independent conditions requires specific metabolic adaptations to cope with energy and oxidative stress^39,40,49,55^. We therefore hypothesized that floating cells will show a metabolic phenotype markedly different from the attached cells. Indeed, we found several changes in the overall comparison of floating versus attached cells. This was especially true for FloatMetDG vs MetDG, while the metabolic profile of FloatHiDG did show a number of similarities to HiDG. Unexpectedly, metabolic profiles of the detached floating cells were more similar to control cells than their attached counterparts. While we did note a trend towards increased percentage of apoptotic or dead cells in FloatHiDG and FloatMetDG compared to HiDG and MetDG, respectively, the difference was too small to possibly explain the changes in metabolite levels. PolyHEMA also exhibited a metabolic profile similar to that of CTRL, with some significantly altered metabolites (tryptophan, proline, xanthosine, cytidine, fructose-1,6-bisphosphate). This suggests that cell detachment itself leads to a partial adaptation of the metabolic profile of FloatMetDG or FloatHiDG back towards that of the untreated control cells with some important changes.

Despite the altered metabolite levels in floating cells, the effects of metformin and 2DG on the glycolytic and OxPhos ATP production remained essentially unchanged. A trend towards increased ATP production was observed in all floating cells (Fig. 6). The total ATP production of FloatHiDG and FloatMetDG was comparable to CTRL. This could be a consequence of strong AMPK activation in floating cells (unpublished observation), consistent with other studies^14,56^. These findings are corroborated by relative ATP levels. Previous research has shown AMP-independent AMPK activation in anchorage independent conditions^56^. Our results support this and suggest that cells detachment might be an additional mechanism that allows cancer cells to better adapt to energy stress.

In addition, NADPH generation and shuttling between mitochondria and cytoplasm is one of the key metabolic adaptations to growth in an anchorage-independent state^40,49^. NADPH levels were significantly higher in FloatHiDG versus HiDG and overall in floating vs. attached cells. We have also found that detached cells upregulate glucose-6-phosphate dehydrogenase, the first enzyme of PPP (unpublished observation). Overall, this seems to support the notion that PPP is upregulated in the floating cells as has previously been shown for cells growing in anchorage independent conditions^39^. Cell detachment might also play some role in adapting to altered redox balance or ROS induced by metformin and 2DG treatment, though this will have to be confirmed by future studies.

Furthermore, cells growing in anchorage independent conditions use reductive carboxylation of glutamine-derived α-ketoglutarate to compensate for the reduced glucose uptake, support anaplerotic reactions and balance reactive oxygen species (ROS)^40,49,55,57^. Importantly, a significant decrease in intracellular glutamine levels in FloatMetDG and FloatHiDG compared to MetDG and HiDG, respectively, was observed in our study. This indicates increased consumption of glutamine, consistent with reductive carboxylation as a metabolic adaptation of detached cancer cells^40,49,55,57^. This could result from increased *PPARGC1A* mRNA expression in floating cells (discussed in more detail in subchapter “Stem-like phenotype of detached cells”), as PGC-1α has been shown to promote glutamine metabolism in estrogen receptor positive breast cancer^58^. However, as glutamate and α-ketoglutarate levels did not show as clear a trend, we cannot exclude changes in other glutamine consuming reactions (eg. transaminations), though metformin has been shown to inhibit glutaminase^48^. No alteration in glutamine metabolism was observed for polyHEMA, suggesting that strong AMPK activation might be enough to support the survival of detached MDA-MB-231 cells in the absence of metabolic drugs^14^.

Amongst other salient changes in floating cells was the altered FA metabolism, where individual FA were decreased in the floating cells. This is in agreement with AMPK activation discussed above, as AMPK inhibits acetyl-coenzyme A carboxylase, a key enzyme in FA synthesis. As FA synthesis consumes NADPH, this inhibition could further help detached cells maintain the NADPH pool and balance ROS. Altered lipid metabolism has recently been shown to play a role in avoiding anoikis and breast cancer cell metastasization to the brain tissue *in vivo*^19,42,57^. Together with our results, this highlights the role of FA metabolism in survival in anchorage independent conditions. We also observed changes in pyrimidine and one-carbon cycle metabolites in floating versus attached cells, suggesting they might support anchorage independent growth. However, these could be only secondary to the changes in energy and redox metabolism.

### Metabolic changes induced by metformin as potential anticancer therapy

As the precise mechanism of action of metformin in the context of potential anti-cancer therapy is still debated, a secondary objective of our study was to elucidate the metabolic changes of metformin in breast cancer cells. In line with published research^22,24–26,32^, we found that metformin, at the concentration used to partially mimic in vivo accumulation with long-term exposure, completely suppressed OxPhos ATP production and lowered ATP levels. To compensate, cells increased glycolysis, in line with our previous work where proliferation was unchanged when sufficient glucose is available^51^. The increase in glycolytic rate resulted in depletion of early glycolysis and PPP metabolite levels, which could drive other observed metabolic changes. Metformin also significantly impacted FA metabolism by increasing levels of four FA (myristic, margaric, nonadecenoic and eicosenoic acid) compared to CTRL. This is in agreement with a recent study by Lord et al. on primary breast cancer cells demonstrating that metformin can suppress FA oxidation, leading to their accumulation^32^. In regards to the redox balance, no clear shift from NAD to NADH typically seen in previous studies^24–26^ was observable. Conversely, an increase in aspartate levels was observed, consistent with lower NADH levels.

Metformin seemed to impact purine metabolism as a whole, as indicated by a trend towards lower levels of NAD, NADH and NADPH, as well as adenine and adenine nucleotides, guanosine and guanine nucleotides. These lower levels are in line with previous studies where metformin affected nucleotides^22,24,25,31^. As purine synthesis requires one-carbon units, it is linked to the folate metabolism. Reports of metformin as an antifolate drug impacting one-carbon metabolism showed increases in homocysteine levels in cancer cells^22^. Our results instead showed decreased homocysteine and increased methionine levels, indicating increased homocysteine methylation. Our results also suggest a possible effect of metformin on the purine degradation pathway. A trend of increased levels of metabolites in late stages of purine degradation (xanthine, xanthosine and uric acid) was detected, while inosine remained unchanged (Supplementary Fig. S7), possibly indicating increased degradation of purines. The purine salvage pathway has come into the spotlight as a potential anti-cancer target in recent years, allowing for the assumption that the anti-cancer effect of metformin might be in part mediated by this pathway^59^.

Metformin also impacted pyrimidine nucleosides, increasing uridine and decreasing cytidine levels. This might be linked to increased glutamine levels seen in Met, as the interconversion of UTP to CTP requires glutamine, and metformin has been shown to inhibit glutaminase^48^. Increased levels of most other AA were also observed, in line with previous studies^24–26^. This might be one of the adaptations allowing the cells to maintain their proliferation with metformin treatment, but which would need further investigations.

### Stem-like phenotype of detached cells

In the process of detachment, cells change their morphology from a spread and elongated to a spherical shape, taking on an even more mesenchymal appearance. As studies have shown that cancer cells growing in anchorage-independent conditions express stemness markers^17,60^, we hypothesized that detached MDA-MB-231 cells might exhibit a more stem-like phenotype. Analyzing the expression of breast cancer stem cell markers CD44 and CD24, we found that over 95% of control MDA-MB-231 cells express CD44 but not CD24, thus exhibiting a breast cancer stem-like cell phenotype. This is consistent with the ability of MDA-MB-231 cells to form metastases in vivo^17,20^. Treating the cells with 2DG and/or metformin or growing them on poly-HEMA did not change the percentage of CD44+/CD24− cells and no differences between floating and attached cells were observable.

The metabolism of cancer stem cells is dependent on the balance between c-MYC and PGC-1α^61^. The *MYC* mRNA levels were lower in HiDG and MetDG as well as FloatHiDG, FloatMetDG and polyHEMA, but no apparent changes between floating and attached cells were detectable. On the other hand, the *PPARGC1A* mRNA levels did show a trend towards higher levels in FloatMetDG and polyHEMA, while the results for FloatHiDG were less clear. While we could not confirm an increase in mitochondrial mass in floating cells compared to their attached counterparts, we observed a clear trend towards higher mitochondrial mass compared to CTRL in both HiDG and MetDG as well as FloatHiDG and FloatMetDG. As stem-like cells growing in anchorage-independent conditions require increased mitochondrial biogenesis^60^, the increased mitochondrial mass could be one of adaptations supporting cell detachment. Overall, although we found no difference in breast cancer stem cell marker expression, the detached cells showed trends potentially indicative of transcriptional and mitochondrial adaptations to a more stem-like metabolic phenotype. This suggests the potential role of detached cells in metastasis formation, but further work is needed to validate these findings.

### Study limitations

Accurately quantifying metabolites in cell lines requires enough cells per sample. In our study, for several treatments, lower cell number per membrane due to significantly suppressed proliferation (Fig. 1b) and cell detachment make metabolites with already reduced concentrations technically difficult or impossible to detect. To partially alleviate the problem, cell seeding density was increased and two membranes were pooled in sample preparation for MetDG and HiDG to reduce the technical artefacts to a minimum. While this approach does somewhat alter the starting conditions between the samples, it also minimizes the differences in cell number (and therefore nutrient consumption) at the point of sampling.

To eliminate a possible bias by using different cell harvesting and extraction methods for floating and attached cells, the boiling ethanol extraction method (so far not used for suspension type cells^62^) was used for all samples. Similar harvesting times between 20 and 30 s were achieved for both cell types in order to prevent a potential metabolic influence of extraction time. The similar metabolite levels detected in poly-HEMA samples compared to control are a proof-of-principle for the selected metabolite extraction method, since a method-bias would introduce major changes in the metabolic profiles. The harvesting methods used also do not discern between live and dead or apoptotic cells. However, as cell viability remained high and in a similar range to control even in the samples with floating cells (see Fig. 1), it could not introduce major bias in the results. In the present study, we used cell culture medium with physiological 1 g/L (5.6 mM) glucose concentration and daily medium change to closely control nutrient levels in order to approximate in vivo conditions as much as possible. As the harvesting method necessitates exposure of cells to atmospheric oxygen levels, we ran the entire experiment at 21% oxygen, which could potentially result in somewhat different metabolite levels compared to in vivo physiological oxygen concentration (around 5%). However, characterizing cell detachment at both oxygen concentrations, we found no qualitative differences in the process between the two oxygen concentrations (Figs. 1d,e).

The used metabolomics method is not suitable for flux analysis in this setting, thus determination of individual pathway contributions to metabolite pools is not feasible. To overcome this shortcoming and complement the results from the metabolic profiling, as well as gaining additional information about OxPhos rate, glycolysis rate and ATP production, Seahorse flux analysis was conducted.

Altogether, we present in depth analysis of the metabolomics profiling in MDA-MB-231 cells in the attached and floating populations. In order to understand if the observed metabolic alterations retain a similar pattern for different breast cancer cell lines we would have to expand the study to other cell lines, such as MCF-7 ER + cell line that partially exhibit cell detachment after MetDG treatment. However the resulting lower viability of detached cells would present a challenge for the metabolomics analysis.

## Conclusions

In this study, we present metabolic profiling results of attached and detached MDA-MB-231 breast cancer cells treated either with 2DG, metformin or with both compounds. We found that metformin induced a distinct change in the metabolic profile of MDA-MB-231 cells both in the overall metabolic phenotype and at individual metabolite levels, consistent with previously published studies. The most pronounced changes were elevated levels of amino acids and FA, as well as metabolites linked to the energy, redox and nucleotide metabolism.

We expected to find a specific metabolic profile responsible for cell detachment. The detachment inducing treatments of metformin + 0.6 mM 2DG and 4.8 mM 2DG resulted in similar metabolic profiles in the attached MDA-MB-231 cells. Since these profiles did not extensively differ from the profile of cells treated with metformin alone (where no cell detachment is observable), the metabolic changes responsible for cell detachment were not unequivocally identifiable. The results suggest the process of cell detachment is a result of a specific effect of 2DG that is not directly linked to inhibition of glycolysis. While the observed metabolic changes seem necessary, they are themselves not sufficient for cell detachment.

The survival of cancer cells in the anchorage independent conditions requires appropriate metabolic adaptations. We therefore expected the floating metformin + 2DG and 4.8 mM 2DG treated cells to have a metabolic profile even more different from the untreated control cells. Our results instead indicate that floating cells were more metabolically similar to the control cells than attached cells treated with metformin + 0.6 mM 2DG or 4.8 mM 2DG. These findings suggest that the process of cell detachment has to be supported with metabolic alterations that compensate energy stress induced by treatment with metformin and/or 2DG. We indeed found common alterations in all cells growing in the anchorage independent conditions compared to the group of all the attached cells, namely in fatty acids, NADPH and amino acids, especially glutamine. This indicates that reductive carboxylation of glutamine-derived α-ketoglutarate and AMP-independent AMPK activation likely play a role in supporting the survival of detached cells, in line with published literature^17,39,40,56^. As the metabolic profile of detached cells is similar to control cells, cell detachment could be a form of adaptation to energy stress^15,40,56^. Since cancer cells have to detach from the primary tumor and survive in anchorage independent condition, the presented results are important for better understanding of metabolic alterations during cell detachment.

## Materials and methods

### Cell culture and treatment

MDA-MB-231 cells were acquired from ATCC and maintained in complete RPMI 1640 medium (Genaxxon Bioscience, Ulm, Germany) supplemented with 10% FBS, 2 mM glutamine and 4.5 g/L glucose. For all experiments, MDA-MB-231 cells were grown at 37 °C and atmospheric oxygen levels in RPMI 1640 medium supplemented with 10% FBS, 2 mM glutamine and 1 g/L glucose unless indicated otherwise.

For the attached population, 325,000 MDA-MB-231 cells were seeded on Lumox 50 membranes (Sarstedt AG& Co, Nümbrecht, Germany) in complete RPMI medium with 4.5 g/L glucose. For samples with significant viable cell detachment, seeding density was increased to 475,000 cells per membrane and two membranes were seeded per sample to achieve comparable cell numbers in the pooled final sample.

After 24 h, cells were washed with 0.9% NaCl, the medium replaced with complete RPMI medium with 1 g/L glucose and treated with 0.6 mM 2DG, 4.8 mM 2DG, 5 mM metformin or 5 mM metformin plus 0.6 mM 2DG for 48 h, with medium renewal after 24 h.

For the floating populations, 215,000 cells per well were plated on 6-well plates in complete RPMI medium with 4.5 g/L glucose. After 24 h, the cells were washed with 0.9% NaCl, their medium replaced with the complete RPMI medium with 1 g/L glucose and treated with 4.8 mM 2DG or 5 mM metformin plus 0.6 mM 2DG for 48 h with medium renewal after 24 h. For anchorage-independent growth condition, 600,000 MDA-MB-231 cells were grown for 48 h on poly-hydroxyethylmetacrylate (poly-HEMA) coated Petri dishes for 48 h with fresh medium added after 24 h.

The effect of glucose concentration and cell density on cell viability and cell number was studied in our previous study^51^. We have shown that cell density does not effect viability and cell number using the same cell culture medium and treatments, if medium is daily renewed daily.

### Metabolite extraction

Metabolite extraction was performed 48 h after treatment start as previously described^63^. For attached cells, medium was aspirated, the Lumox membrane was cut and immediately dip washed three times in 5.6 mM ^13^C_6_-glucose (Sigma Aldrich, 389374) in 0.9% NaCl at 37 °C. Samples were then immediately quenched by pushing the membrane in 15 mL centrifuge tubes containing 3 mL 75% ethanol + 25% 1.5 M ammonium acetate preheated to 85 °C. Tubes were incubated for 2 min at 85 °C and immediately placed on dry ice. Each Lumox membrane was harvested in 30 s or less from medium aspiration to quenching. Samples were harvested in the same time sequence as the prior medium change to eliminate any confounding effects.

For samples with floating cells and cells grown on poly-HEMA, the filtration protocol for suspension cells was used as previously described^62^. Medium containing floating cells was collected from two 6-well plates with a pipette and transferred onto an ethanol pre-wetted standard PTFE filter (Porafil, 47 mm, 0.22 µM PTFE) placed in a filter funnel and immediately filtered. Immediately upon completion of filtration, cells were washed with 4 mL of 5.6 mM ^13^C_6_-glucose in 0.9% NaCl prewarmed to 37 °C. After washing, filter was removed from the funnel and pushed into 15 mL centrifuge tubes containing 3 mL 75% ethanol + 25% 1.5 M ammonium acetate preheated to 85 °C. Following 2 min of incubation at 85 °C, tubes were immediately placed on dry ice. The entire harvesting process from cell suspension filtration start to quenching in boiling ethanol was completed in under 30 s and samples were harvested in the same time sequence as the prior medium change to eliminate any confounding effects.

After metabolite extraction, samples were dried with nitrogen gas. For 4.8 mM and 5 mM metformin + 0.6 mM 2DG treated samples with attached cells and 5 mM metformin + 0.6 mM 2DG treated samples with floating cells, two tubes were pooled to reach comparable amounts of metabolites. Samples were re- constituted in 100 µL water for LC/MS measurement. A small aliquot from each sample was pooled for quality control (QC) with 20 μL from cell samples and all samples were frozen at − 80 °C until measurement.

### Metabolomic profiling

Samples, blanks (pure H_2_O), QCs, and UltimateMix (UM) were measured in a separate stratified randomized sequence for attached and floating cells and analyzed with a Dionex Ultimate 3000 HPLC (Thermo Fisher Scientific, Waltham, Massachusetts, USA) equipped with a reversed-phase Atlantis T3 C18 pre- and analytical column (Waters, Milford, Massachusetts, USA) with an injection volume of 10 µL as described previously^64–66^. To reduce unwanted metabolite degradation, the measurement was divided into two 24 h batches and only samples for a 24 h batch were freshly thawed and placed into the autosampler at 4 °C. Raw data was converted into mzXML (msConvert, ProteoWizard Toolkit v3.0.5)^66^, and detected m/z of known metabolites were identified using PeakScout^63,67,68^ with a reference list containing accurate mass and retention times.

Technical variability was 14% median relative standard deviation (RSD) in the QC samples for 53 multivariate and univariate analyses (MVA_UVA) metabolites and yielded further 46 univariate analysis (UVA) metabolites (see Supplementary Table 1). The four main classes in the ontology-distribution for the MVA_UVA metabolites classified according to the Human Metabolome Database^69^ were nucleotides and analogues (18 metabolites), amino acids, peptides and analogues (17 metabolites), carbohydrates and conjugates (5 metabolites), and metabolites related to energy metabolism (7 metabolites).

### Real-time ATP production rate assay

For attached cells, MDA-MB-231 cells were plated on Seahorse XFe24 cell culture microplates at 20,000 cells per well. in complete RPMI 1640 medium (2 mM glutamine, 0 mM pyruvate, 10% FBS) with 4.5 g/L glucose. After 24 h, cells were washed with isotonic NaCl solution and the medium replaced with complete RPMI 1640 medium (2 mM glutamine, 0 mM pyruvate, 10% FBS) with 1 g/L or 0 g/L glucose as indicated. Cells were treated with 5 mM metformin, 0.6 mM or 4.8 mM 2-deoxy-D-glucose (2-DG), or a combination of these for 48 h, with a medium renewal after 24 h. After 48 h of treatment, the medium was replaced with the appropriate RPMI 1640-based Seahorse XF Glycolytic Rate Assay medium (2 mM glutamine, 1 mM HEPES, 1 g/L glucose) equilibrated to pH 7.4, with the same concentrations of metformin and 2DG as the treatment media for 45 min at 37 °C without CO_2_.

For detached cells, MDA-MB-231 cells were plated on 6-well plates at 160,000 cells per well for control cells and 215,000 cells per well for cells treated with 4.8 mM 2DG or 5 mM metformin + 0.6 mM 2DG. After 24 h, cells were washed with isotonic NaCl solution and the medium replaced with complete RPMI 1640 medium with 1 g/L glucose and treated with 4.8 mM 2DG or 5 mM metformin + 0.6 mM 2DG for 48 h with daily medium change. For cells grown on poly-HEMA, MDA-MB-231 cells were seeded in complete RPMI medium with 1 g/L glucose on poly-HEMA coated 6-well plates at 215,000 cells per well for 48 h with daily addition of medium. After treatment, detached cells population were collected and attached cells were detached with trypsin. Both cell populations were spun down and resuspended in Seahorse XF RPMI 1640-based Seahorse XF Glycolytic Rate Assay medium (2 mM glutamine, 1 mM HEPES, 0 mM pyruvate, 1 g/L or 0 g/L glucose) equilibrated to pH 7.4 and plated on Seahorse cell culture microplates covered with CellTak® at 50,000 cells in 0.1 mL per well. Plates were spun down at 200 g for 1 min and incubated for 15 min at 37 °C without CO_2_, after which 0.4 mL of medium was added and after additional 30 min of incubation at 37 °C without CO_2_.

For both the attached and detached cells, the oxygen consumption rate (OCR) and extracellular acidification rate (ECAR) were measured on the Seahorse XFe24 analyzer and the ATP production rate from glycolysis and oxidative phosphorylation determined according to Seahorse Real-Time ATP Rate Assay protocol.

### Cell detachment

MDA-MB-231 cells were plated on 6-well plates at 145.000 cells per well for control cells. After 24 h, cells were washed with isotonic NaCl solution and the medium replaced with complete RPMI 1640 medium with 1 g/L glucose and treated with 4.8 mM 2DG or 5 mM metformin + 0.6 mM 2DG for 48 h either at 21% with daily medium change or at 5% with a surplus of medium to avoid exposing the cells to additional oxygen during the medium change. After treatment, the attached and detached cells were collected separately, stained with Trypan Blue and counted, and the percentage of detached cells calculated.

### Flow cytometry

MDA-MB-231 cells were seeded on 12-well culture plates in complete RPMI medium with 4.5 g/L glucose. After 24 h, the cells were washed with isotonic NaCl solution and the medium was changed to complete RPMI with 1 g/L. Cells were treated with 0.6 mM or 4.8 mM 2DG, 5 mM metformin or their combination for 48 h or 72 h with daily medium change. For determination of mitochondrial mass, cells were stained after 72 h treatment with 7.5 μM nonyl-acridine orange (NAO) for 15 min at 37 °C. Cells were then harvested, resuspended in PBS and their fluorescence measured on Attune NxT flow cytometer. For stemness marker expression, cells were harvested after 48 h of treatment by replacing the culture medium with PBS + 2 mM EDTA for 5 min at room temperature and washing them with a pipette. Cells were counted and 100,000 cells were stained with anti-CD24 (PE-conjugated, 311105, Biolegend, San Diego, California, USA) and anti-CD44 (APC-conjugated, 338805, Biolegend) antibody at room temperature for 20 min. Cells were then washed and analyzed on Attune NxT flow cytometer (Thermo Fisher Scientific).

For the percentage of dead and apoptotic cells, MDA-MB-231 cells were seeded on 6-well plates at the same density per cm^2^ as for metabolomics profiling, cultured and treated as described above for 48 h. Cells were harvested, centrifuged and resuspended in annexin staining buffer (10 mM HEPES, 140 mM NaCl, 2.5 mM CaCl_2_, pH 7.4) and stained with annexin V-Alexa Fluor® 488 conjugate (Thermo Fischer Scientific, A13201) for 15 min at room temperature. 0.15 mM Propidium iodide (PI) was added immediately before the measurement and the cells were analysed on Attune NxT flow cytometer.

### Quantitative real-time PCR

MDA-MB-231 cells were seeded on 6-well culture plates in complete RPMI medium with 4.5 g/L glucose. After 24 h, the cells were washed with isotonic NaCl solution and the medium was changed to complete RPMI with 1 g/L glucose. Cells were treated with 0.6 mM or 4.8 mM 2DG, 5 mM metformin or a combination for 48 h. After treatment, attached and detached cell populations were harvested and washed twice in ice-cold PBS and snap frozen in liquid nitrogen. Total mRNA was isolated using TRI Reagent (Sigma-Aldrich, St. Louis, Missouri, USA) and reverse transcribed with High-Capacity cDNA Reverse Transcription Kit (Thermo-Scientific). Quantitative real-time PCR was performed on QuantStudio 12 K Flex Real-Time PCR System (ThermoFisher Scientific) with LightCycler 480 SYBR Green I Master reaction mix (Roche, Basel, Switzerland). The following primers (all Sigma-Aldrich) were used to detect gene-specific mRNAs (target, forward/reverse primer): *MYC*, 5’-TACCCTCTCAACGACAGCAG-3’/5’- AGCCTGCCTCTTTTCCACA-3’; *PPARGC1A*, 5’-AAAAGCCACAAAGACGTCCC-3’/5’-TGTTGGTTTGGCTTGTAAGTGT-3’; *B2M*, 5’-TTCTGGCCTGGAGGCTATC-3’/5’-TCAGGAAATTTGACTTTCCATTC-3’. The *B2M* gene expression was used as housekeeping control. Quantification was performed using the comparative Ct (-ΔΔCt) method.

### Statistical analyses

Statistical analysis for metabolomics was performed with R (R Core Team, v3.4.1) and TibcoSpotfire (v7.5.0). All metabolites were controlled for their analytical quality as described previously^63^ and graded into two classes: (I) suitable for multivariate analysis and univariate analysis (MVA_UVA) and (II) suitable for univariate analysis (UVA). Quality assessment was done according to Vogel et al^63^, and a separate control for outliers due to sequence position, time point of measurement, replicate number, cell count, date of sampling, measurement batch and sample extraction events was conducted via Principal Component Analysis (PCA). To correct for cell number differences and reduce technical variability, median QC normalization was performed and the data log_10_-transformed.

Kolmogorov–Smirnov and Brown-Forsythe Levene tests were used to discern normality and homoscedasticity of data distribution. The log_10_-transformed, normalized data were normally distributed according to the Kolmogorov Smirnov test (66.7% of all metabolites were normally distributed) and homoscedastic according to the Brown-Forsythe Levene-type test (98.0% of all metabolites were homoscedastic). Six of the samples was excluded due to a technical problem during the sample injection during HPLC–MS measurement. All values and additional information are reported in Supplemental Sheet Excell file.

PCA analysis was performed, centered and scaled to unit variance (R function prcomp). Missing values were imputed by regularized expectation–maximization (R function impute PCA, estim_ncpPCA). For ANOVA analysis the treatment was taken as the fixed factor (simple ANOVA; R function gls, missing values were not imputed). For specific group comparisons, pairwise post-hoc tests (R function lsmeans) were conducted. Benjamini–Hochberg corrected *p* values (R function *p* adjust) are listed in the supplementary Excell file, while in the main paper *p* values without correction are given for specific a priori determined hypotheses.

MANOVA was calculated in RStudio (V 3.6.0) using the function #manova (test-Pillai) (data log-median-QC normalized). Metabolites were assigned to pathways as described in Supplementary Table 3.

Heatmap analysis was done in Spotfire (V 7.5.0) using median of log-median-QC normalized metabolite levels per treatment group and metabolic pathway. Dendrograms were calculated automatically using UPGMA clustering with Euclidian distance measure and average ordering value and row interpolation and column average for missing value replacement.

For extracellular flux analysis, flow cytometry and quantitative real-time PCR, one-way ANOVA with Dunnett’s post-hoc test was used to test statistical significance of differences using GraphPad Prism software.

## Supplementary Information

Supplementary Information.
