# Supplementary material for: Metabolic profiling of attached and detached metformin and 2-deoxy-D-glucose treated breast cancer cells reveals adaptive changes in metabolome of detached cells
Source: Sci Rep. 2021 Nov 1;11:21354. doi: 10.1038/s41598-021-98642-0 (PMC8560930; doi:10.1038/s41598-021-98642-0)
Supplement: Supplementary file 1 — Supplementary Information. [file 41598_2021_98642_MOESM1_ESM.pdf]

Supplementary information

MVA UVA Metabolites		UVA Metabolites	
Anhydrosorbitol	Glutamine	Hexose mono-phosphate	FFA C19:2 Nonadecadienoic acid
Guanine	Histidine	Lactic acid	Glycerol-3-phosphate
Hexose	Phenylalanine	p-Cresyl sulphate	Phosphate
Homocysteine	AMP	Pentose	Proline
Phosphogluconic acid	GMP	Taurine	Valine
Lysine	ADP	Thymol	Glutamic acid
Methionine	CTP	Adenosylhomocysteine	FFA C8:0 Caprylic acid
NAD	cAMP	Xanthine	Arginine
NADH	Pantothenic acid	FFA C12:0 Lauric acid	Fructose-1,6-bisphosphate
NADP	Xanthosine	FFA C14:0 Myristic acid	Valerenic acid
NADPH	Guanosine	FFA C15:0 Pentadecylic acid	FFA C12:0 Dodecanedioic acid
Acetyl-CoA	Inosine	FFA C12:1 Lauroleic acid	Adenine
Oxoglutaric acid	Pyridoxine	FFA C14:1 Myristoleic acid	Cytidine
Pyroglutamic acid	Phosphoglucono-D-lactone	FFA C16:1 Palmitoleic acid	Hydroxy-benzoic acid
Tyrosine	Hydroxyisocaproic acid	FFA C19:1 Nonadecenoic acid	Acetylcarnitine
Uric acid	N-Acetyl-glutamic acid	FFA C20:1 Eicosenoic acid	Aminoadipic acid
Uridine	N-acetyl-L-alanine	FFA C12:2 Dodecadienoic acid	Histidinol
Uridine monophosphate	Oxidized glutathione	FFA C16:2 Palmitolinoleic acid	N-acetyl-L-ornithine
FFA C17:0 Margaric acid	UDP-N-acetyl-glucosamine	FFA C18:2 Linoleic acid	Phthalic acid
FFA C14:2 Tetradecadienoic acid	N-Acetyl-L-aspartic acid	FFA C9:0 Pelargonic acid	Sedoheptulose-1,7-bisphosphate
FFA C13:2 Tridecadienoic acid	Aspartic acid	FFA C10:0 Capric acid	Coenzyme A
Tryptophan	Leucine and Isoleucine	FFA C11:0 Undecylic acid	Phosphohydroxypyruvate
Ornithine	Cytidine monophosphate	FFA C13:0 Tridecanoic acid	Methyl-2-oxovaleric acid
GDP	UDP-D-Glucose		
ATP	Gluconic acid		
GTP	Glycine		
Asparagine			

Supplementary Table S1: List of MVA_UVA and UVA metabolites.

**Glycolysis**	**Pentose Phosphate Pathway**	**Glycosylation intermediates**	**Glutamine metabolism**	**Redox metabolism**
Lactic acid	6-Phosphoglucono-D-lactone	UDP-N-acetyl-glucosamine	a-Ketoglutarate	Oxidized-glutathione
Hexose mono-phosphate	Sedoheptulose-1-7-bisphosphate	UDP-D-Glucose	Glutamic acid	NAD
Glycerol 3-phosphate	6-Phosphogluconic acid		Glutamine	NADH
Hexose	Pentose			NADP
Fructose 1.6-bisphosphate				NADPH
**Beta Oxidation**	**Unsaturated fatty acids**	**Nucleotides**	**Purine Metabolism**	**Pyrimidine Metabolism**
Acetyl-CoA	FFA C14:2 (Tetradecadienoic acid)	ADP	ADP	Cytidine monophosphate (CMP)
FFA C10:0 (Capric acid)	FFA C12:1 (Lauroleic acid)	AMP	AMP	Uridine
FFA C12:0 (Lauric acid)	FFA C14:1 (Myristoleic acid)	ATP	ATP	Uridine monophosphate (UMP)
FFA C14:0 (Myristic acid)	FFA C16:1 (Palmitoleic acid)	cAMP	cAMP	Cytidine
FFA C8:0 (Caprylic acid)	FFA C16:2 (Palmitolinoleic acid)	GDP	GDP	CTP
	FFA C19:1 (Nonadecenoic acid)	GMP	GMP	Glutamine
FFA C17:0 (Margaric acid)	FFA C20:1 (Eicosenoic acid)	GTP	GTP	
FFA C11:0 (Undecylic acid)	FFA C13:2 (Tridecadienoic acid)	Cytidine monophosphate (CMP)	Aspartic acid	
FFA C13:0 (Tridecanoic acid)	FFA C12:2 (Dodecadienoic acid)	Uridine monophosphate (UMP)	Glutamine	
FFA C15:0 (Pentadecylic acid)	FFA C19:2 (Nonadecadienoic acid)	CTP	Glycine	
FFA C9:0 (Pelargonic acid)			Glutamic acid	
Acetylcarnitine			Guanine	
			Guanosine	
			Inosine	
			Uric acid	
			Xanthosine	
			Adenine	
			Xanthine	
**Other AA**	**One carbon metabolism**	**Urea cycle**	**Vitamins, Coenzymes, Cofactors**	
Asparagine	Methionine	Ornithine	Pantothenic acid	
Histidine	Adenosylhomocysteine	Arginine	Pyridoxine	
Lysine	Homocysteine	Aspartic acid	Coenzyme A	
N-Acetyl-L-aspartic acid				
Phenylalanine				
Tryptophan				
Tyrosine				
Aminoadipic acid				
Proline				
Valine				
Leucine, Isoleucine				
N-acetyl-l-alanine				

Supplementary Table S2: Classification of metabolites per metabolic pathways for heat map analysis.

Glycolysis	Pentose Phosphate Pathway	Purine nucleotides	Beta Oxidation
Lactic acid	6-Phosphoglucono-D-lactone	ADP	Acetyl-CoA
Hexose mono-phosphate	Sedoheptulose-1-7-bisphosphate	AMP	FFA C10:0 (Capric acid)
Hexose	6-Phosphogluconic acid	ATP	FFA C12:0 (Lauric acid)
Fructose 1.6-bisphosphate	Hexose mono-phosphate	GDP	FFA C14:0 (Myristic acid)
		GMP	FFA C8:0 (Caprylic acid)
		GTP	FFA C17:0 (Margaric acid)
			FFA C11:0 (Undecylic acid)
**One carbon metabolism**	**Pyrimidine Metabolism**	**Purine Metabolism**	FFA C13:0 (Tridecanoic acid)
Methionine	Cytidine monophosphate (CMP)	Guanine	FFA C15:0 (Pentadecylic acid)
Adenosylhomocysteine	Uridine	Guanosine	FFA C9:0 (Pelargonic acid)
Homocysteine	Uridine monophosphate (UMP)	Inosine	
	Cytidine	Uric acid	
	CTP	Xanthosine	
		Adenine	
		Xanthine	

Supplementary Table S3: Metabolite classification according to metabolic pathways for MANOVA.

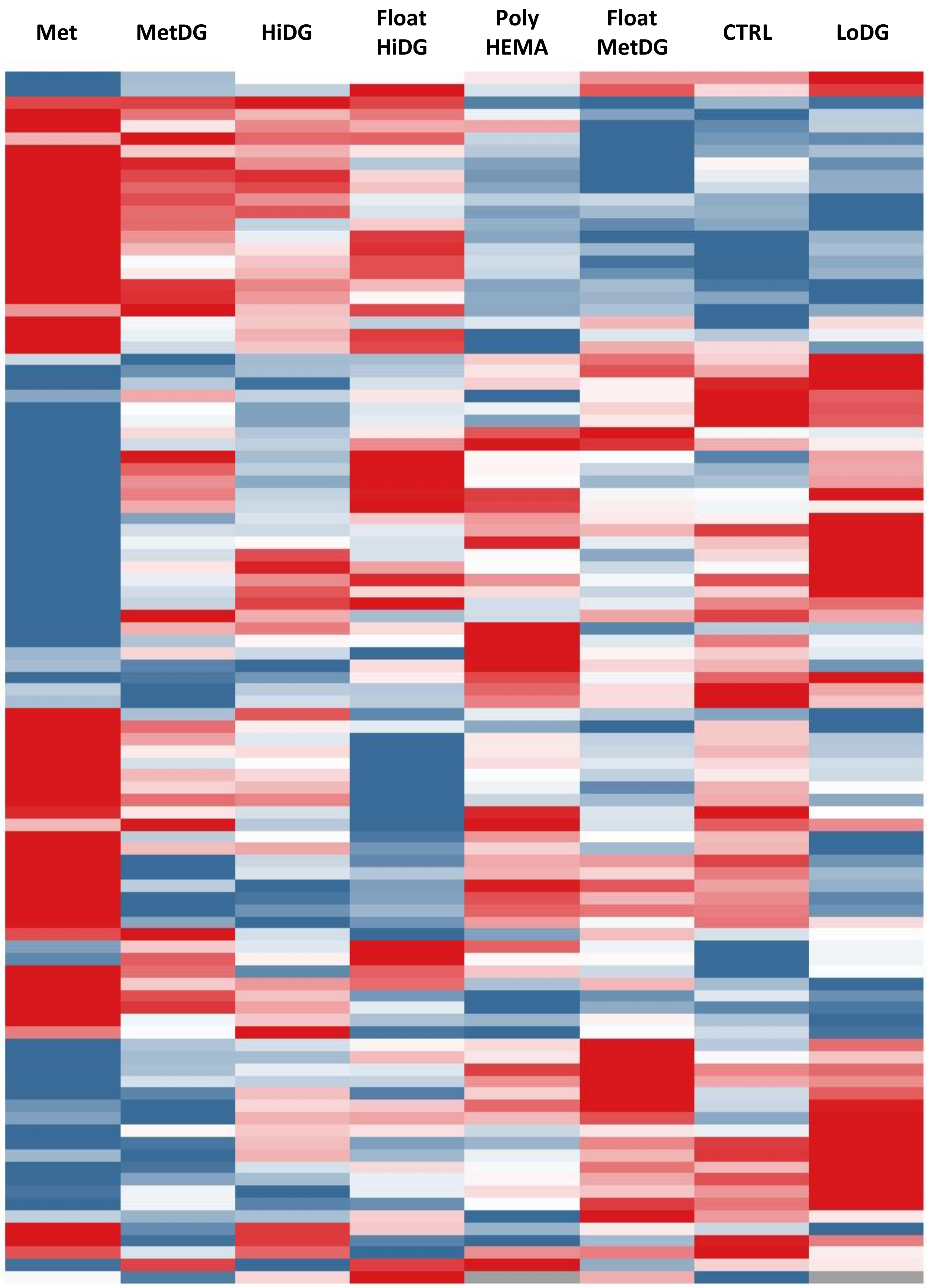

Supplementary Figure S1: Heat map clustering for individual metabolites. For each metabolite and treatment group, the log-median-QC normalized value was used. Red – maximum levels, blue – minimum levels.

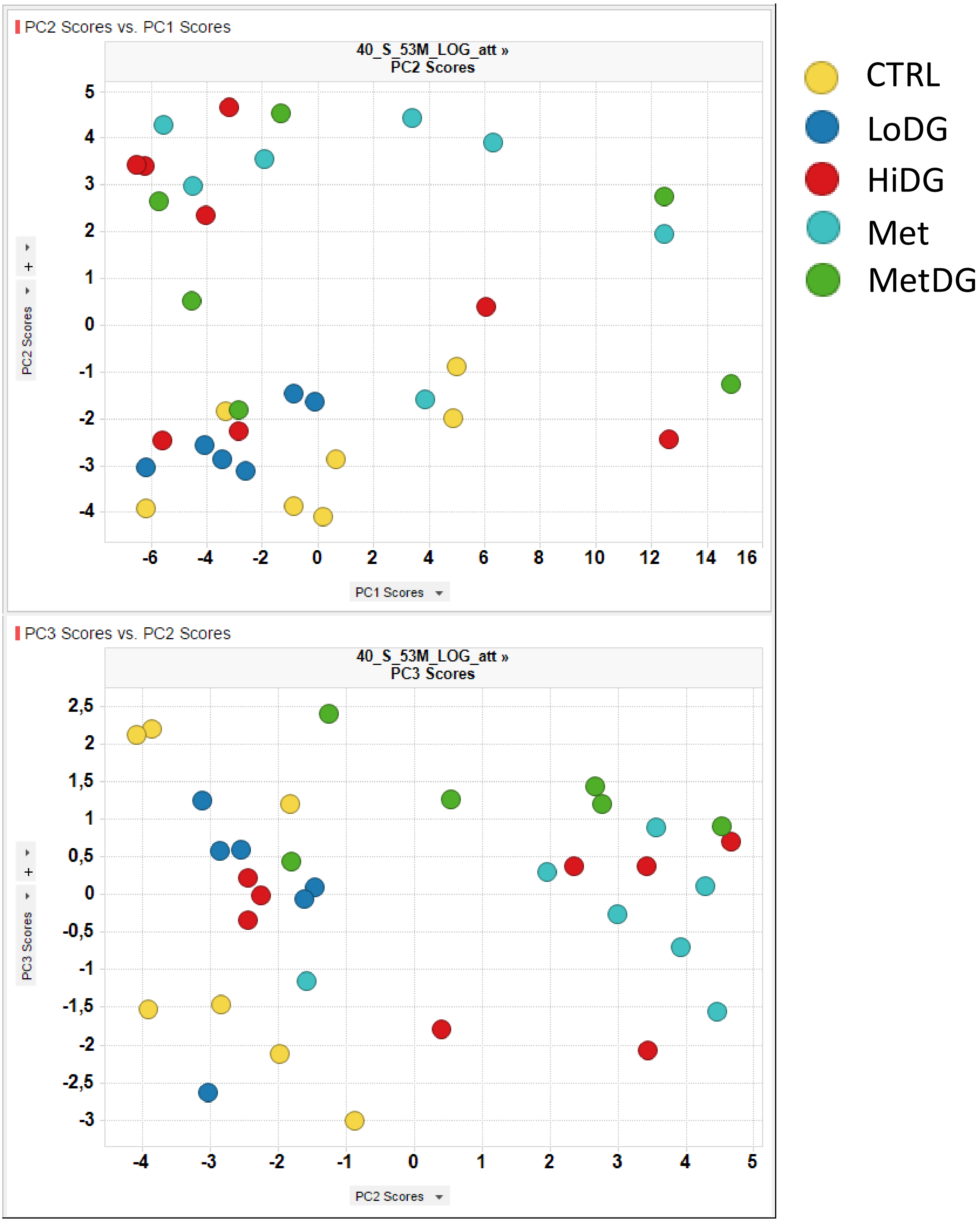

Supplementary Figure S2: PCA scores for attached cells for the first 3 PCA components. Left scores plot shows PC1 vs. PC2, while right scores plot shows PC2 vs. PC3. The QC-normalized data was log-transformed and data points are colored according to treatment. The attached population showed group separations of Met and MetDG from CTRL in the PCA. HiDG also separated from the CTRL in a bimodal way, with some samples clustering closer to CTRL, while showing closer PCA relations to Met. LoDG had a modest effect, while LoDG treated-cells showed no clear separation from CTRL.

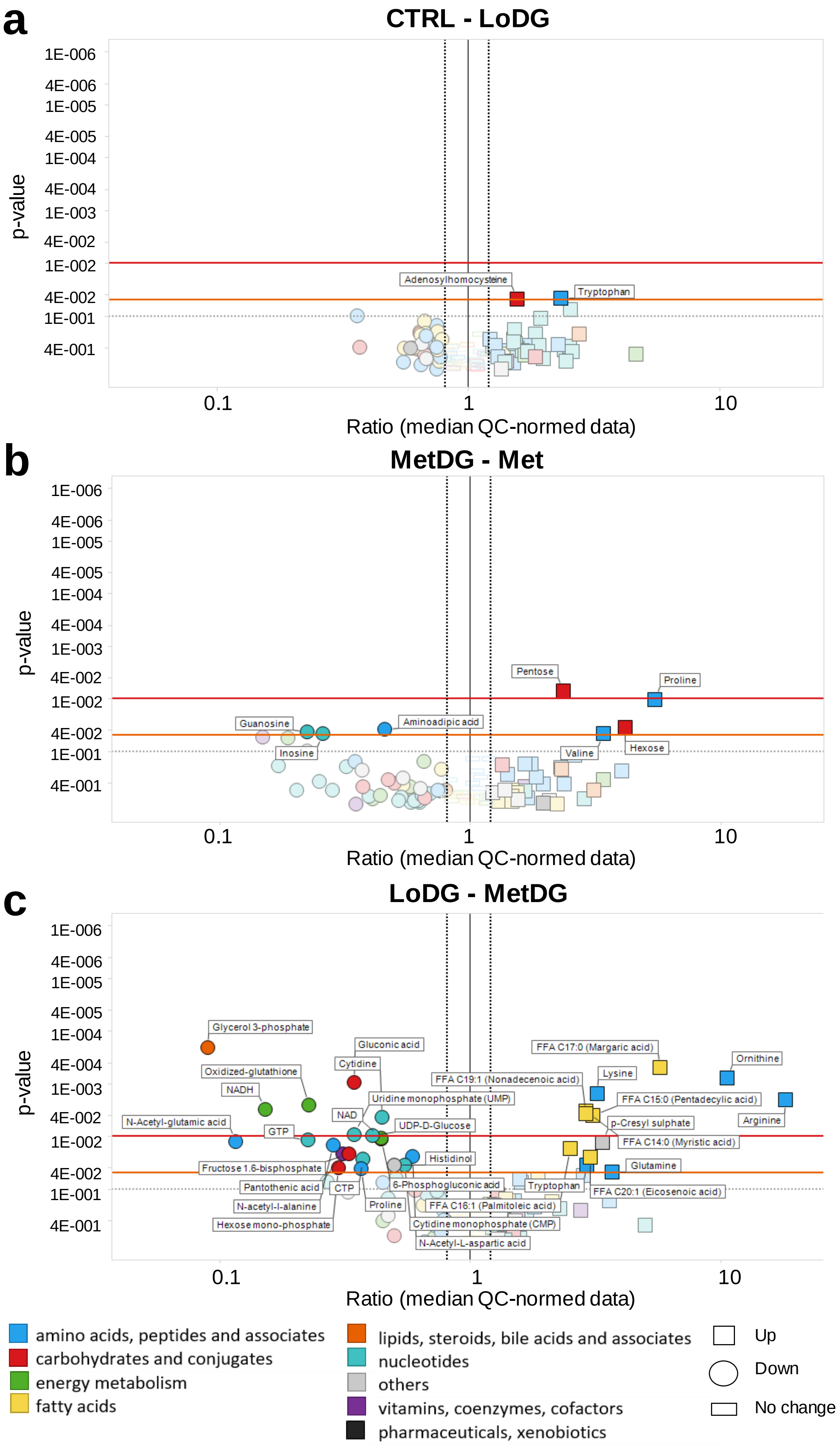
a
CTRL - LoDG
p-value
Ratio (median QC-normed data)
Adenosylhomocysteine
Tryptophan
b
MetDG - Met
p-value
Ratio (median QC-normed data)
Pentose
Proline
Guanosine
Aminoadipic acid
Inosine
Valine
Hexose
c
LoDG - MetDG
p-value
Ratio (median QC-normed data)
Glycerol 3-phosphate
Gluconic acid
Cytidine
Oxidized-glutathione
NADH
N-Acetyl-glutamic acid
GTP
NAD
Uridine monophosphate (UMP)
UDP-D-Glucose
FFA C17:0 (Margaric acid)
Ornithine
Lysine
FFA C19:1 (Nonadecenoic acid)
FFA C15:0 (Pentadecylic acid)
Arginine
p-Cresyl sulphate
FFA C14:0 (Myristic acid)
Glutamine
Histidinol
Fructose 1.6-bisphosphate
Pantothenic acid
CTP
N-acetyl-l-alanine
Hexose mono-phosphate
6-Phosphogluconic acid
Tryptophan
FFA C20:1 (Eicosenoic acid)
Proline
FFA C16:1 (Palmitoleic acid)
Cytidine monophosphate (CMP)
N-Acetyl-L-aspartic acid
amino acids, peptides and associates
carbohydrates and conjugates
energy metabolism
fatty acids
lipids, steroids, bile acids and associates
nucleotides
others
vitamins, coenzymes, cofactors
pharmaceuticals, xenobiotics
Up
Down
No change

Supplementary Figure S3: Volcano plots of metabolite levels in the attached cells for LoDG compared to CTRL (A) and MetDG compared to Met (B) and LoDG (C). Metabolites on the left side of the black line were decrease while those on the right side of the black line were increased in LoDG compared to CTRL (**A**), Met compared to MetDG (**B**) or MetDG compared to LoDG (**C**). Y-axis: p-values (inverse log scale); x-axis: metabolite ratios (median QC normalized data, log-scaled). Left black dotted line (0.8) - ratio of 0.8; right black dotted line (1.2) - ratio of 1.2. The red line denotes a p-value of 0.01, the orange line denotes a p-value of 0.05 and the grey line denotes a p-value of 0.1. Metabolites are colored according to the HMDB classes. Metabolites with p-value bellow 0.05 and metabolite ratio >1 or <0.8 are named. Metabolites are marked with a circle for decrease and a square for increase in LoDG compared to CTRL (**A**), Met compared to MetDG (**B**) or MetDG compared to LoDG (**C**).

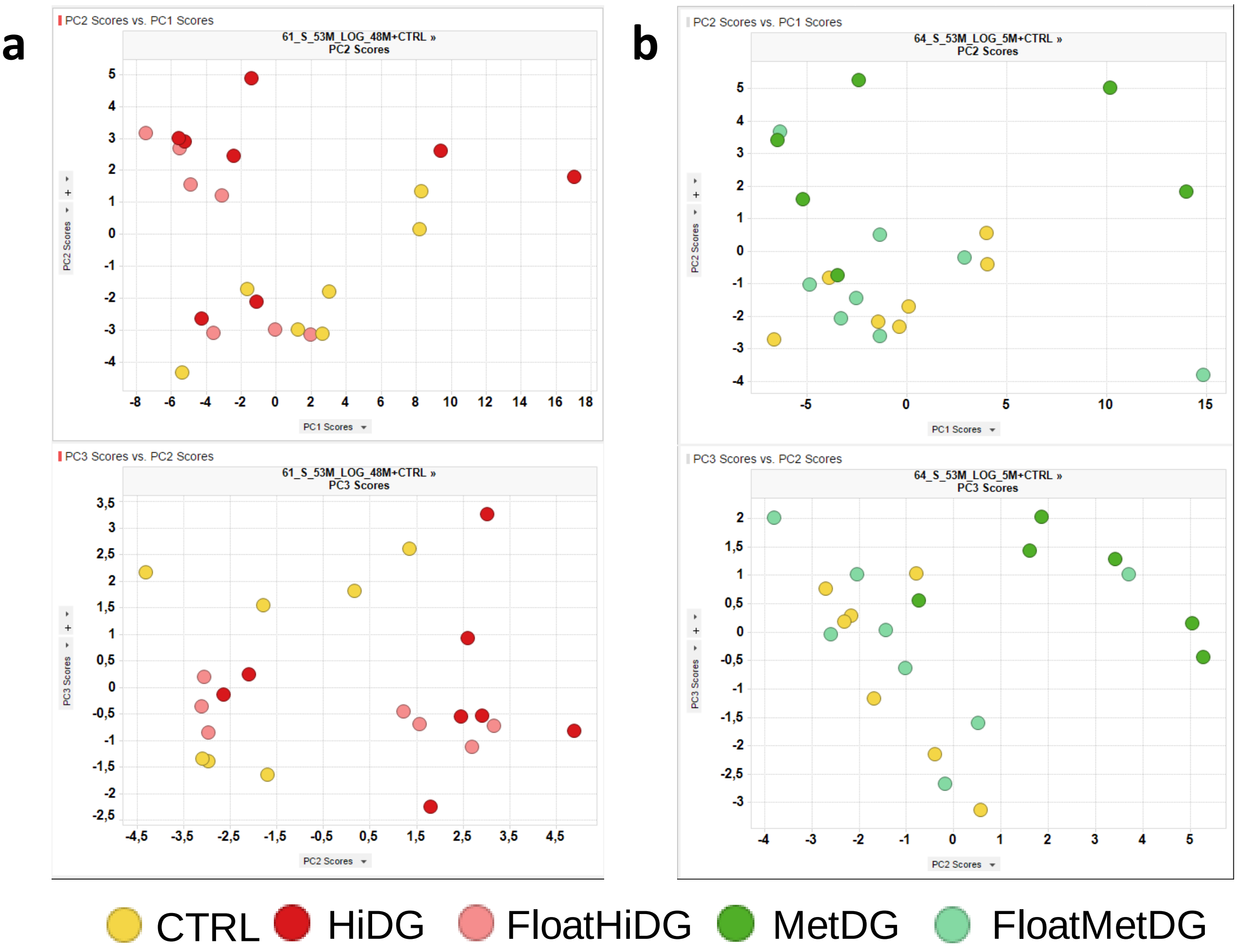

Supplementary Figure S4: PCA scores for CTRL and metformin + 2DG (A) or 4.8 mM 2DG (B) treated cells for the first 3 PCA components. Top graphs show PC1 vs. PC2, while bottom graphs show PC2 vs. PC3. The QC-normalized data was log-transformed. Data points are colored according to treatment (yellow – control; green – metformin + 2DG; red – 4.8 mM 2DG) and attachment status (light red and green – floating; dark red and green - attached).

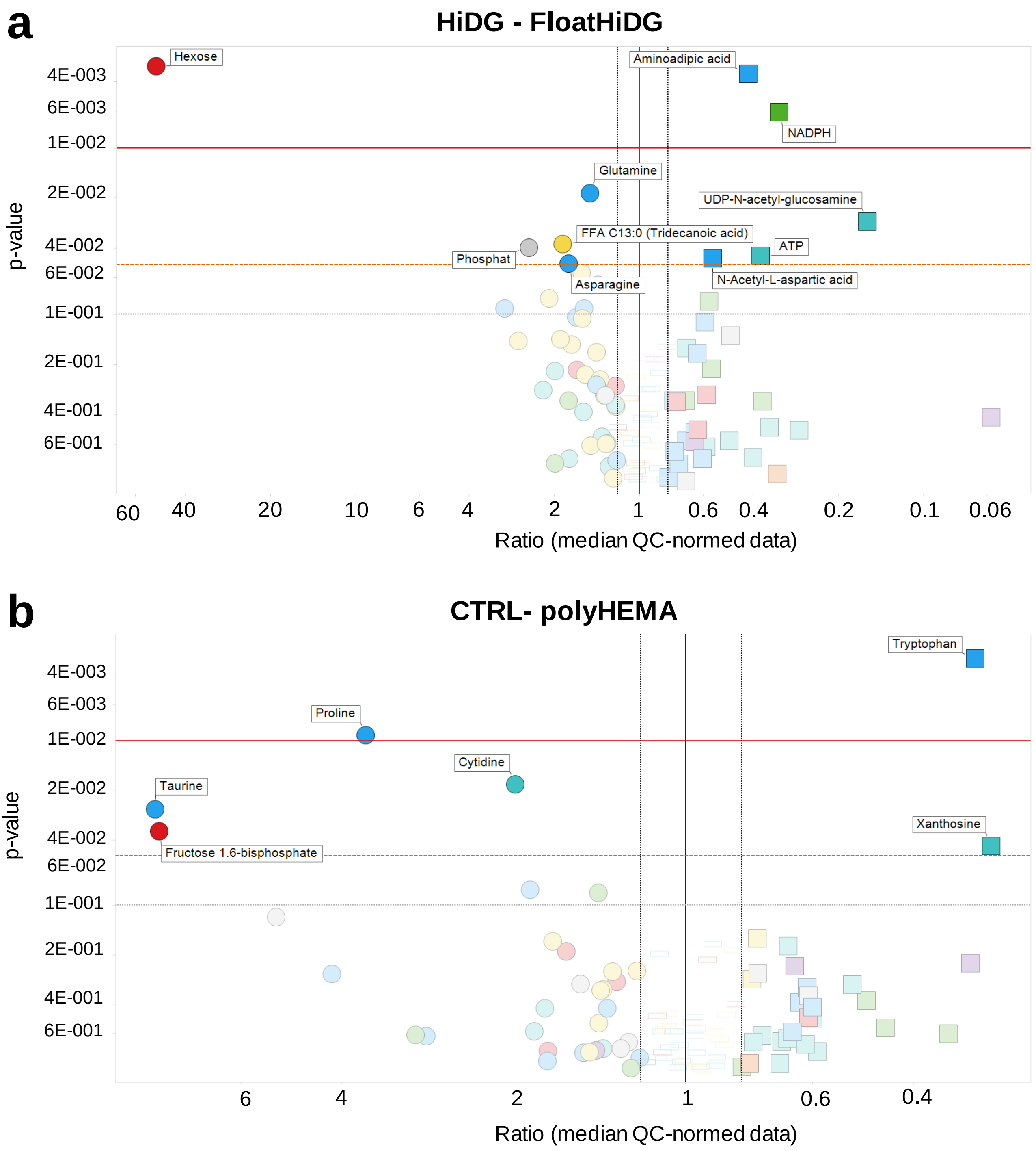

Supplementary Figure S5: Volcano plots of metabolite levels in the floating cells for FloatHiDG compared to HiDG (A) and polyHEMA compared to CTRL (B). Metabolites on the left side of the black line were decreased while those on the right side of the black line were increased in FloatHiDG compared to HiDG (**A**) or polyHEMA compared to CTRL (**B**). Y-axis: p-values (inverse log scale); x-axis: metabolite ratios (median QC normalized data;). Please note the scale is different for (**A**) and (**B**). Left black dotted line (0.8) - ratio of 0.8; right black dotted line (1.2) - ratio of 1.2. The red line denotes a p-value of 0.01, the orange line denotes a p-value of 0.05 and the grey line denotes a p-value of 0.1. Metabolites are colored according to the HMDB classes. Metabolites with p-value bellow 0.05 and metabolite ratio >1 or <0.8 are named. Metabolites are marked with circle for decrease and square for increase in FloatHiDG compared to HiDG (**A**) or polyHEMA compared to CTRL (**B**)

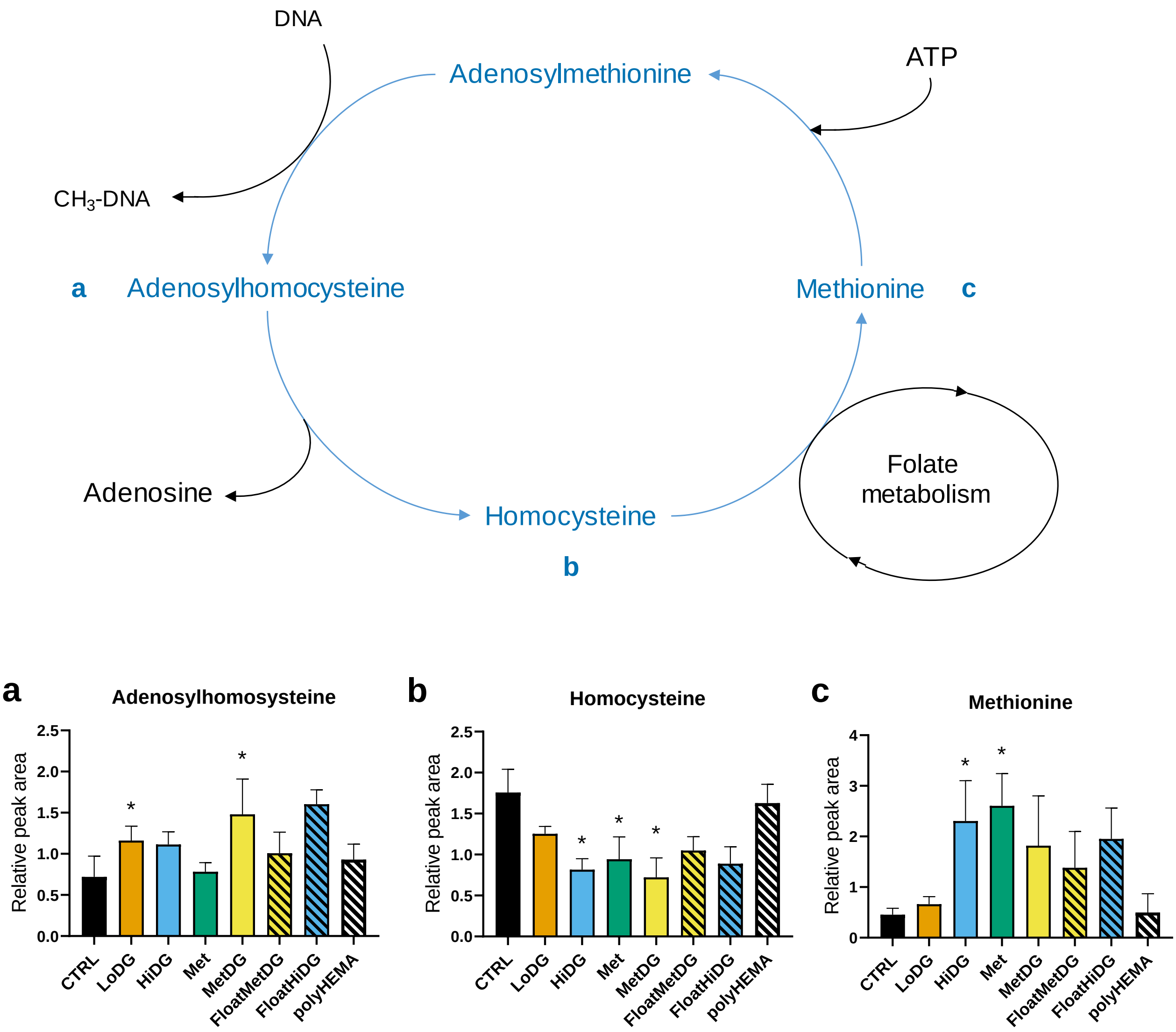

Supplementary Figure S6: Metabolite levels in one carbon cycle. Metabolite levels were normalized and mapped to one carbon cycle to show the metabolic reactions in the cell. Met, MetDG and HiDG decreased ($p<0.05$) homocysteine, while Met and HiDG increased ($p<0.05$) methionine levels compared to CTRL. There were no apparent differences between floating and attached cells. Samples are color-coded and samples with floating cells are hatched. All graphs show mean relative normalized metabolite peak areas +/- SE. *$p<0.05$, **$p<0.01$. ***$p<0.001$, ****$p<0.0001$ versus CTRL unless otherwise indicated.

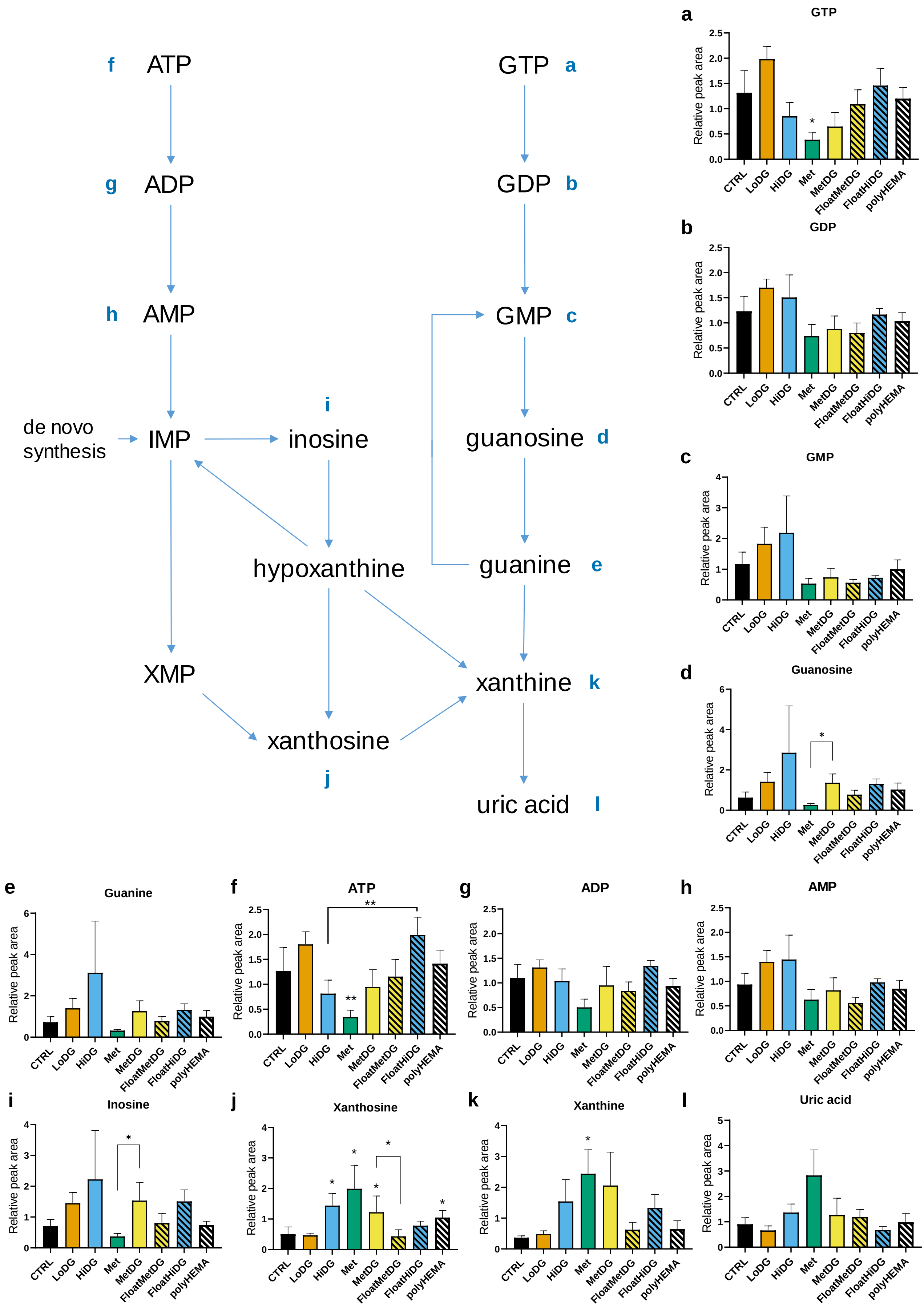
f ATP
g ADP
h AMP
de novo synthesis
IMP
i inosine
hypoxanthine
XMP
j xanthosine
GTP a
GDP b
GMP c
guanosine d
guanine e
xanthine k
uric acid l
a GTP
b GDP
c GMP
d Guanosine
e Guanine
f ATP
g ADP
h AMP
i Inosine
j Xanthosine
k Xanthine
l Uric acid
Relative peak area
CTRL
LoDG
HiDG
Met
MetDG
FloatMetDG
FloatHiDG
polyHEMA

Supplementary Figure S7: Purine metabolism. Metabolite levels were normalized and mapped to one carbon cycle to show the metabolic reactions in the cell. Metformin treatment strongly influenced purine metabolism, showing a clear trend towards lower guanine and adenine nucleotides while late-stage purine degradation metabolites trended higher. This might point to the possible effect of metformin on the purine degradation pathway. This effect was not shared with 2DG, as in HiDG most purine metabolites (including adenine and guanine nucleotides) trended higher. Interestingly, treatment with 0.6 mM 2DG in addition to metformin (MetDG) partially reversed the effect of metformin on most metabolites. There were some differences between floating and attached population for xanthosine. Samples are color-coded and samples with floating cells are hatched All graphs show mean relative normalized metabolite peak areas +/- SE. *p<0.05, **p<0.01. ***p<0.001, ****p<0.0001 versus CTRL unless otherwise indicated.

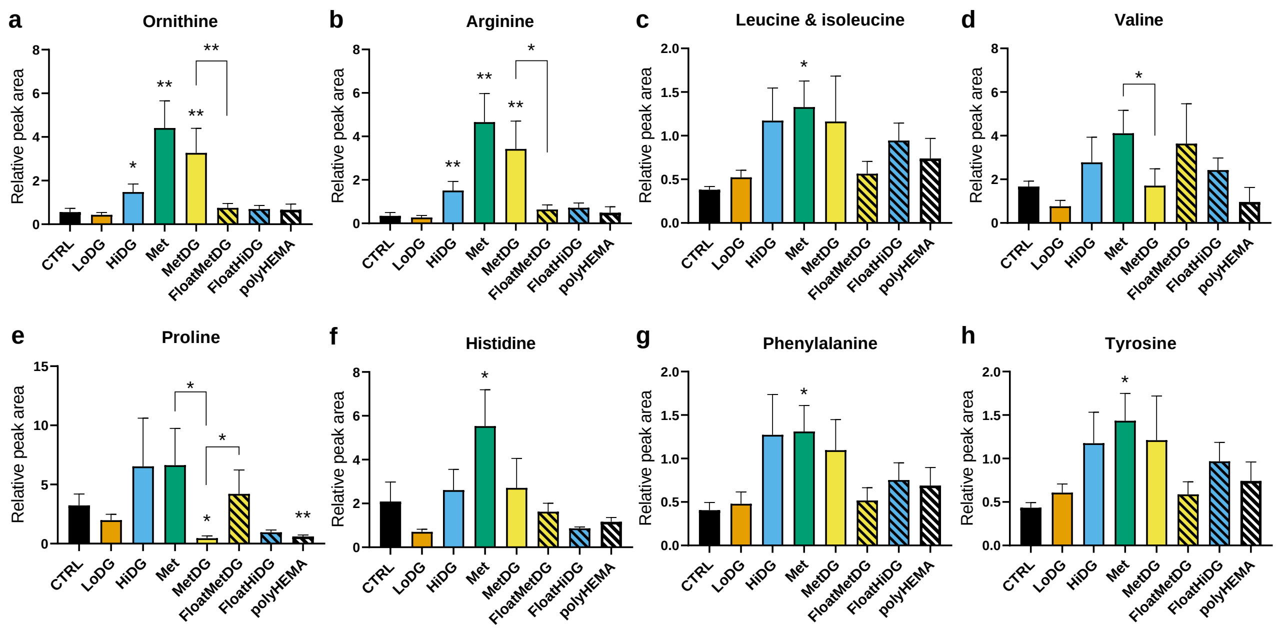

Supplementary Figure S8: Amino acid metabolism. Samples are color-coded and samples with floating cells are hatched. Most amino acids levels were increased with metformin treatment compared to CTRL. MetDG showed similar trends to Met for most amino acids except histidine, valine and proline. Proline levels were actually lower in MetDG compared to both CTRL and Met. HiDG also showed a trend towards higher amino acids vs. CTRL. Levels of amino acids (except for proline) in floating cells were similar CTRL. All graphs show mean relative normalized metabolite peak areas +/- SE. Please note the difference in y-axis scales. *p<0.05, **p<0.01. ***p<0.001, ****p<0.0001 versus CTRL unless otherwise indicated.
